# Association mapping for protein, total soluble sugars, starch, amylose and chlorophyll content in rice

**DOI:** 10.1186/s12870-022-04015-8

**Published:** 2022-12-29

**Authors:** D. K. Nayak, S. Sahoo, S. R. Barik, P. Sanghamitra, S. Sangeeta, E. Pandit, K. R. Reshmi Raj, N. Basak, S. K. Pradhan

**Affiliations:** 1 ICAR-National Rice Research Institute, Cuttack, Odisha 753006 India; 2 College of Agriculture, OUAT, Bhabaneswar, Odisha 751003 India; 3 Fakir Mohan University, Balasore, Odisha 756020 India

**Keywords:** Association mapping, Protein content, Soluble sugars content, Starch content, Amylose content, Chlorophyll content, Rice

## Abstract

**Background:**

Protein, starch, amylose and total soluble sugars are basic metabolites of seed that influence the eating, cooking and nutritional qualities of rice. Chlorophyll is responsible for the absorption and utilization of the light energy influencing photosynthetic efficiency in rice plant. Mapping of these traits are very important for detection of more number of robust markers for improvement of these traits through molecular breeding approaches.

**Results:**

A representative panel population was developed by including 120 germplasm lines from the initial shortlisted 274 lines for mapping of the six biochemical traits using 136 microsatellite markers through association mapping. A wide genetic variation was detected for the traits, total protein, starch, amylose, total soluble sugars, chlorophyll a, and chlorophyll b content in the population. Specific allele frequency, gene diversity, informative markers and other diversity parameters obtained from the population indicated the effectiveness of utilization of the population and markers for mapping of these traits. The fixation indices values estimated from the population indicated the existence of linkage disequilibrium for the six traits. The population genetic structure at K = 3 showed correspondence with majority of the members in each group for the six traits. The reported QTL, *qProt1*, *qPC6.2*, and *qPC8.2* for protein content; *qTSS8.1* for total soluble sugar; *qAC1.2* for amylose content; *qCH2* and *qSLCHH* for chlorophyll a (Chl. a) while *qChl5D* for chlorophyll b (Chl. b) were validated in this population. The QTL controlling total protein content *qPC1.2*; *qTSS7.1*, *qTSS8.2* and *qTSS12.1* for total soluble sugars; *qSC2.1*, *qSC2.2*, *qSC6.1* and *qSC11.1* for starch content; *qAC11.1*, *qAC11.2* and *qAC11.3* for amylose content; *qChla8.1* for Chl. a content and *qChlb7.1* and *qChlb8.1* for Chl. b identified by both Generalized Linear Model and Mixed Linear Model were detected as novel QTL. The chromosomal regions on chromosome 8 at 234 cM for grain protein content and total soluble sugars and at 363 cM for Chl. a and Chl. b along with the position at 48 cM on chromosome 11 for starch and amylose content are genetic hot spots for these traits.

**Conclusion:**

The validated, co-localized and the novel QTL detected in this study will be useful for improvement of protein, starch, amylose, total soluble sugars and chlorophyll content in rice.

**Supplementary Information:**

The online version contains supplementary material available at 10.1186/s12870-022-04015-8.

## Background

Rice is life and principal staple food crop for the large global population. But, the protein content in rice grain is low. Protein is highly required for plant growth and development. It takes part in numerous biochemical reactions in the body and acts as hormone, antibody, performs transport and storage of nutrients and many more functions. Protein content also affects the eating and cooking quality of rice [1]. Enhancement of protein content through breeding is effective, economical and reasonably an easier way to combat protein malnutrition [2]. Total soluble sugars (sucrose, glucose and fructose) and starch play important role for signalling; maintain the overall structure and growth of plants and response to the stresses [3, 4]. Total soluble sugars (TSS) influence organoleptic quality of seeds and are the key factors for development of fresh and sweet flavours [5]. Rice kernel is rich in carbohydrate which constitute > 80% starch. Protein content in the rice kernel is about 7–8% [6]. Starch profiles of rice are controlled by a complex genetic system (multiple quantitative trait loci). Amylose content (AC) is considered as the indirect index of major physical and chemical attributes of the starch [7]. Starch and protein are basic metabolites of seed that influence the eating and cooking qualities, nutritional qualities and health benefits of grains [8]. Amylose and amylopectin are two different types of starch found in rice endosperm of which amylose content mainly affects the eating and cooking qualities of rice [9]. The percentage of starch, amylose, protein and total soluble sugar content (TSS), are the key determinant biochemical factors which affects seed quality [10].

Higher chlorophyll content in rice varieties produce more dry matter and grain yield than low chlorophyll containing genotypes. Chlorophyll content (CC) is used in rice breeding programs as an effective index for high photosynthetic efficiency [11]. The Chl. a and b content of leaves are the main pigments of photosynthesis in the chloroplasts. They are responsible for the absorption and utilization of the light energy influencing photosynthetic efficiency [12]. Continuous efforts are being paid by rice breeders for improvement of these traits. However, significant and stable improvement has not been achieved due to the role of many genes/quantitative trait loci which are also affected by environment. Many QTL controlling seed protein content in rice grain have been reported from the mapping studies in rice [2, 13, 14]. Few QTL controlling chlorophyll content have been reported from the genetic analysis studies [15–17]. Detection of QTL for controlling the TSS in rice grain have been reported in few publications [18, 19]. In addition, very few reports on mapping of starch [20–22] and amylose content [23, 24] are available in rice.

Association mapping is an effective approach to detect genes/QTL for complex traits with a wide genetic pool through marker-trait association analysis. Naturally occurring variations can be exploited to detect QTL that regulate such traits in rice through association mapping. The study of genetic diversity and structure is helpful to recognize the population behaviour. Population structure (Q) with relative kinship (K) analyses were used to check the panel population composition for linkage disequilibrium (LD) mapping. The marker-trait association based on both the generalized linear model (GLM) and mixed linear model (MLM) were estimated and have been shown to perform better than other model analysis. For easy improvement of eating, cooking, nutritional qualities and chlorophyll content, we need robust molecular markers and also validation the reported QTL for improvement of these traits through marker-assisted breeding. Therefore, this mapping study will provide novel QTL and validation of these reported target QTL including use in marker-assisted breeding. In this study, the main target was to detect the candidate genes/QTL for total protein, total soluble sugars, starch, amylose and chlorophyll content in rice by genotyping with 136 simple sequence repeat (SSR) markers covering all the chromosomes.

## Method

### Plant materials

**Fig. 1:**
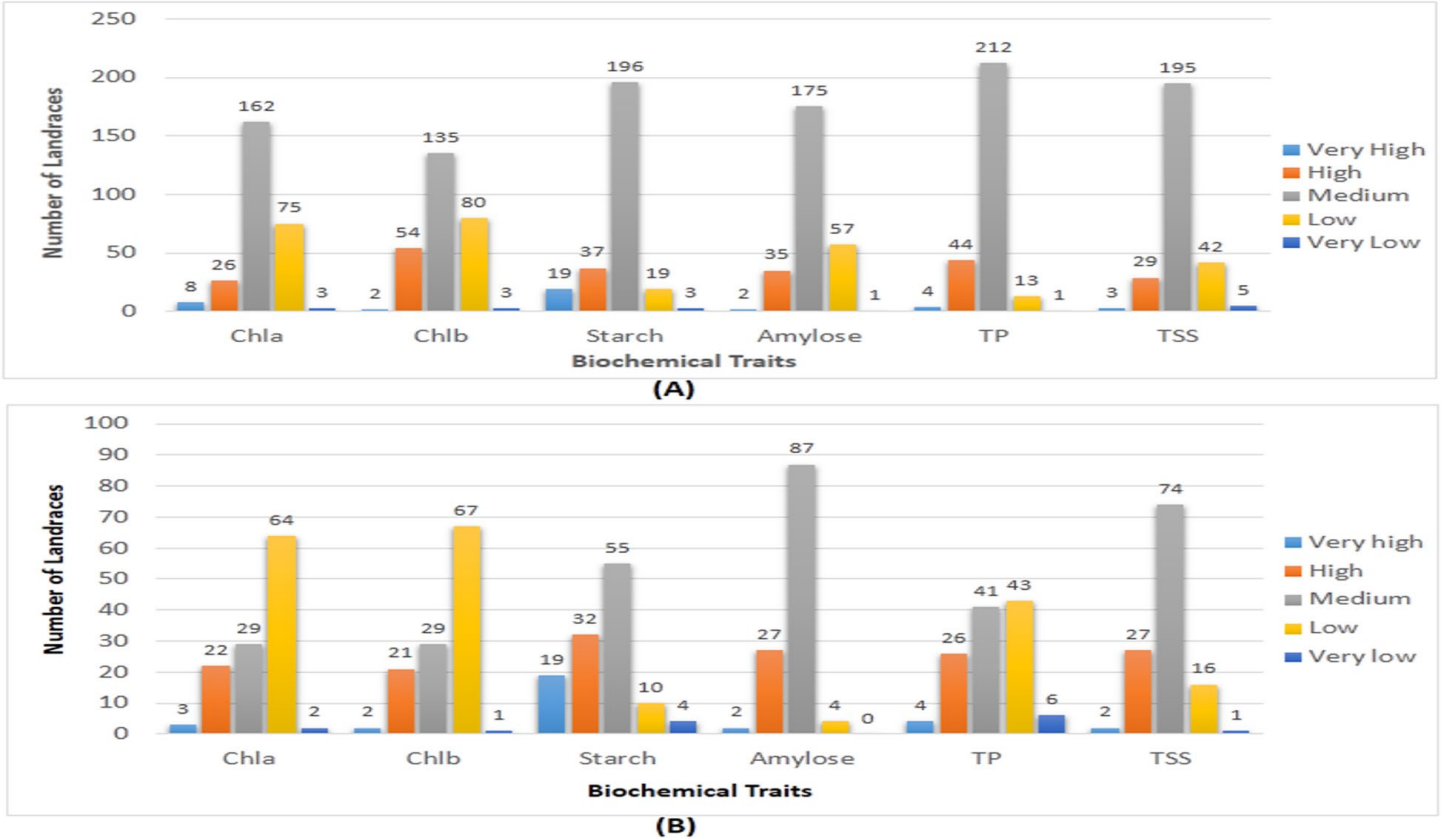
Frequency distribution of germplasm lines for very high, high, medium, low and very low for chlorophyll a, chlorophyll b, starch, amylose, total protein and total soluble sugars estimated (**A**) from 274 rice landraces (**B**) from 120 landraces present in the panel population

A set of 274 diverse rice germplasm lines were collected from Gene Bank of ICAR-NRRI, Cuttack were used in the study (Supplementary Table 1; Fig. 1A). The set was constituted by the germplasm collections from Assam, Madhya Pradesh, Kerala, Odisha and Manipur. For breaking of seed dormancy the harvested seeds were stored for 3 months for the estimation of biochemical traits like total protein content (TP), starch, amylose, total soluble sugars, chlorophyll a and b. A representative panel population was developed by including 120 germplasm lines from the initial shortlisted 274 lines for mapping of the six biochemical traits using 136 microsatellite markers through association mapping.

### Phenotyping for biochemical traits and statistical analyses

The chlorophyll a and chlorophyll b content were estimated using the leaf samples of 10 days old seedlings by following the procedure suggested by Arnon [25]. Chl. a and Chl. b were expressed in mg/g fresh wt. leaf. Calibrated Near Infrared Spectroscopy (NIRS) was used to estimate starch (%), amylose (%) and protein (%). The NIR was calibrated following the procedure of Bagchi et al. [26]. Various modified partial least square (mPLSs) models corresponding with the best mathematical treatments were identified for starch, amylose and protein content. A total of 15 g dehusked rice grain sample was taken in a small cup (size: inner diameter 66 mm and height 25 mm) and the above traits were measured in calibrated NIR spectroscopy. TSS content was estimated calorimetrically by the Anthrone method [27] and was expressed in percentage. Cropstat software7.0 was used to estimate critical difference (CD) and coefficient of variation (CV %) in the recorded phenotypic data.

### Genomic DNA isolation, PCR analysis and selection of SSR markers

Seeds of panel comprising 120 rice accessions were germinated in the petri plates. After 15 days, leaves were collected and genomic DNA was extracted using CTAB method [28]. The isolated DNA was quantified through gel electrophoresis and PCR analysis was performed using 136 SSR markers covering all the chromosomes (Supplementary Table 2). The reaction conditions were set for denaturation, annealing and extension. The PCR products were separated using 3% agarose. A 50 bp DNA ladder was used to determine the base pair of the amplicons. Electophoresis was performed by running the gel for 4 hr. at 2.5 V/cm and band images were captured using the Gel Documentation System (SynGene). The method for genomic DNA isolation, PCR analysis and selection of SSR marker followed in earlier publications were adopted in this study [29–31].

### Molecular data analysis

For each genotype-primer combination, amplicons were scored for the presence or absence of the amplified products. The data was entered as discrete variables into a binary data matrix. For each SSR locus, the number of alleles (N), observed heterozygosity (H), major allele frequency (A), expected heterozygosity (He), and polymorphic information content (PIC) were estimated by using Power marker V3.25 [32]. A similarity matrix table was generated from the binary data using Jaccard’s coefficients. The cladogram was generated using method of unweighted pair group method arithmatic average (UPGMA) algorithm [33, 34] and was visualized by Treeview 32 software [35]. The population structure, cluster analysis and AMOVA were performed using STRUCTURE 2.3.6, Darwin 5 and GenAlEx 6.5 software, respectively. STRUCTURE was run with the optimal number of groups (K) varying from 1 to 10, with 10 runs for each K value. To determine the true value of K, adhoc statistic ∆K value was followed [36]. Parameters were set to 1,50,000 burn-in periods and 1,50,000 Markov Chain Monte Carlo (MCMC) replications after burn-in with an admixture and allele frequencies correlated model. The procedures followed for the software used were described in previous publications [37–39].

### Association analysis

TASSEL 5 software was used to know the marker-trait association of the six biochemical traits. Two statistical models namely, General linear model and Mixed linear model were used in the TASSEL 5.0 software. The genetic association between phenotypic trait of the rice accessions and SSR makers were determined using the software [40]. Markers which are significantly associated with the traits were identified based on the markers *r*^2^and *p*-values. The false discovery rate (FDR) and adjusted *p-* values (q values) were also calculated. The false discovery rate (FDR) in the association study were computed following the previous publications [37, 41].

## Results

### Phenotyping for protein, total soluble sugars, starch, amylose and chlorophyll content in the rice germplasm lines

**Fig. 2:**
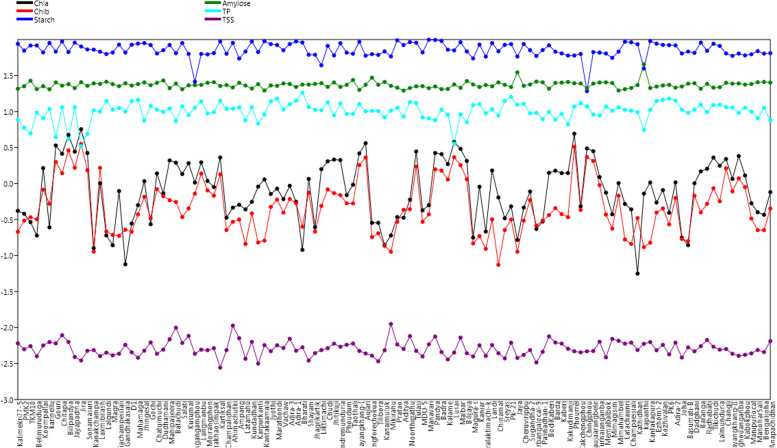
Variation plot for chlorophyll a, chlorophyll b, starch, amylose, total protein and total soluble sugars estimated from the 120 germplasm lines present in the panel population

A total of 274 rice germplasm lines were phenotyped for protein, total soluble sugars, starch, amylose and chlorophyll content (Supplemental Table 1). A representative population was used as panel population which was developed from the original germplasm lines based on the mean phenotypic values of the six traits. Each trait was classified into different phenotypic groups based on the mean estimates of the traits. Phenotyping results for protein, total soluble sugars, starch, amylose and chlorophyll content of the 274 lines showed clear-cut differences among the genotypes (Supplementary Table 1: Fig. 1A). The frequency distribution of the original population was broadly classified into 5 groups for each of the 6 biochemical traits studied (Fig. 1A). A working panel population was constituted by selecting 120 germplam lines from all the phenotypic groups of each trait (Table 1). The estimated mean values of the 6 biochemical traits from the panel population also revealed significant variation among the genotypes for each trait (Table 1; Fig. 1B; Fig. 2). Very high value of  > 15% grain protein content was detected in the landraces, Bharati and Pk-21. In addition, > 12.5% protein was obtained from the germplasm lines Lalgundi, D1, Mahamaga, Langmanbu, Kartiksal, Jyothi, Adira-1, Adira-3, Chudi, Pondremunduria, Sreyas, Cheruvirippu, Kakchengphou, Ezhoml-2 and Kozhivalan. Mikirahu, Batachudi, Chitapa, Kusumal, Ahimachutki, Ampang, Mikirahu, Noorthipathu, Pandya and Malbar showed very high values for total soluble sugars. Very high starch content of > 95% was observed in the landraces Manavari, Pandya, Badra and Kantakapura. Intermediate amylose content is desirable for consumption, but very high content of about 30% and more was noticed in the landraces Kapanthi, Jaya and Chingforechokua. Very high Chl. a content of > 3 mg/g fresh leaf was noticed in the line Jira, Bilipandya, Gauri, Aujari, Lusai and Malbar. Very high content of > 2 mg/g fresh wt. leaf Chl. b was detected in the germplasm lines Jira, Bilipandya, Aujari, Lusai, Phourrel, Chingphou and Phoaujaarangbele (Table 1). The genotypes identified may be useful as donor parents for improvement of these traits in future breeding programs.

**Table 1:** Mean estimates of chlorophyll a, chlorophyll b, starch, amylose, total protein and total soluble sugars content in the panel population containing 120 landraces

Sl. No.	Name of the germplasm	Chl. a (mg/g)	Chl. b (mg/g)	Starch (%)	Amylose (%)	TP (%)	TSS (%)
1	Kalimekri77-5	0.418461	0.213689	87.285	20.73	7.685	0.006039
2	PMK2	0.380092	0.30564	70.33	22.49	5.95	0.004993
3	TKM10	0.291091	0.340809	82.795	26.79	4.955	0.00552
4	Belimuruduga	0.189559	0.318845	82.81	20.53	9.695	0.003981
5	Koompallai	1.642195	0.820699	66.31	22.53	8.05	0.005643
6	Karinellu	0.246103	0.524419	89.745	20.375	10.87	0.006279
7	Gouri	3.384948	1.995411	68.155	25.41	4.43	0.006023
8	Chitapa	2.586631	1.395932	94.125	22.92	11.495	0.007821
9	Bilipandya	4.747542	2.887384	67.165	24.1	4.17	0.006319
10	Jayapadma	2.770007	1.657586	90.715	21.19	11.525	0.003904
11	Jira	5.685392	3.542826	80.215	25.585	3.305	0.003488
12	Sonamasuri	2.656077	1.532689	72.835	22.775	4.88	0.004753
13	Kanakchampa	0.126664	0.11333	72.5	24.62	10.38	0.004853
14	Laxmibilash	1.011124	1.65089	67.78	24.22	10.005	0.004007
15	Lalgundi	0.189861	0.215795	62.985	25.95	14.06	0.004477
16	Magra	0.139128	0.193363	66.175	24.045	10.64	0.004107
17	Gondia Champeisiali	0.786828	0.187396	85.015	22.525	11.2	0.004327
18	Gandhakasala	0.075528	0.228298	66.13	24.715	10.03	0.00575
19	D1	0.278761	0.214976	84.695	22.985	13.94	0.004534
20	Mahamaga	0.50054	0.373229	87.905	24.125	14.565	0.003807
21	Jhingesal	1.07755	0.654155	89.605	25.395	7.47	0.00477
22	Gochi	0.272075	0.329535	84.04	23.24	12.08	0.006219
23	Chatuimuchi	1.381812	0.834547	64.13	25.14	10.6	0.00432
24	Dudhamani	0.728266	0.668823	71.195	27.01	9.93	0.004933
25	Mahipaljeera	2.093718	0.587545	84.09	21.16	11.065	0.006865
26	Batachudi	1.947769	0.554541	64.795	24.495	7.405	0.00994
27	Salati	1.357858	0.342431	89.705	20.41	11.865	0.006106
28	Kusumal	1.916321	0.451783	63.27	23.13	8.915	0.007655
29	Phongangangamphou	1.032898	0.723265	26.045	23.265	11.28	0.004324
30	Langmanbu	1.970748	1.378707	63.195	23.475	13.835	0.004927
31	Moirangphon	1.083463	0.802947	62.275	25.145	9.45	0.004843
32	Chakhaosimpak	0.893333	0.678752	64.885	25.575	9.79	0.005167
33	Kartiksal	2.301049	1.341315	94.195	22.655	14.14	0.002771
34	Champalidhan	0.335878	0.2259	63.57	23.33	10.915	0.00486
35	Ahimachutki	0.462676	0.29343	89.975	21.61	10.94	0.010636
36	Ampang	0.507059	0.31592	54.575	25.055	11.45	0.007105
37	Latamahu	0.437713	0.144814	68.21	22.985	7.585	0.003678
38	Kundadhan	0.557657	0.384152	94.02	20.9	11.46	0.006326
39	Karpurkanti	0.907579	0.151935	67.215	24.04	6.815	0.003151
40	Kantakaamala	1.142496	0.16122	94.82	19.61	9.125	0.005683
41	Jyothi	0.716138	0.47429	86.405	23.06	13.905	0.004694
42	Marathondi	0.849118	0.599011	84.085	22.76	15.09	0.00567
43	Vachaw	0.608423	0.395134	71.225	24.565	10.63	0.005197
44	Adira-3	0.931634	0.609701	86.645	24.125	12.705	0.006965
45	Adira-1	0.55722	0.533002	94.215	21.98	14.21	0.004763
46	Bharati	0.11991	0.250789	91.08	23.705	18.255	0.005316
47	Shayam	1.159831	0.744995	61.54	23.705	11.585	0.003474
48	Jhagrikartik	0.247011	0.215269	61.485	24.215	10.59	0.004407
49	Liktimachi	1.586021	0.489175	43.93	24.76	10.43	0.004857
50	Chudi	2.035862	0.828522	81.685	22.08	13.405	0.005163
51	Jhitikuji	2.150431	0.735869	60.3	25.385	8.85	0.005983
52	Pondremunduria	2.118815	0.690361	88.985	22.03	12.975	0.005406
53	Phoudum	0.69057	0.531774	65.975	23.515	9.29	0.005756
54	Taothali	0.96362	0.529258	63.525	27.39	9.305	0.005986
55	Mayangkhang-I	2.62352	1.807781	93.22	19.97	12.71	0.00473
56	Aujari	3.638041	2.302221	59.76	23.57	10.055	0.004364
57	Chingforechokua	0.285212	0.180568	62.785	29.56	10.225	0.004067
58	Tilibora	0.285145	0.203468	61.21	23.56	10.185	0.003394
59	Kanaimuluk	0.139297	0.136113	68.495	25.8	8.31	0.004837
60	Mikirahu	0.190164	0.112745	58.395	22.735	10.315	0.011206
61	Pratao	0.342026	0.294541	97.805	21.48	11.255	0.005819
62	Aditya	0.335272	0.432	83.92	19.55	8.525	0.005047
63	Noorthipathu	0.595589	0.441051	92.305	21.23	13.51	0.007608
64	Tulasi	2.788855	1.72611	90.385	22.41	13.11	0.004747
65	MDU-5	0.424576	0.293781	66.455	22.535	8.335	0.003961
66	Manavari	0.50054	0.373229	99.495	21.115	8.035	0.005846
67	Pandya	2.655943	1.578489	98.795	22.305	7.595	0.007498
68	Badra	2.560894	1.510666	95.65	20.49	10.58	0.004564
69	Kalame	1.863504	1.139251	72.4	20.61	9.02	0.003608
70	Lusai	3.784023	2.323776	70.795	23.825	3.6	0.004494
71	Malbar	3.02992	1.804037	91.76	21.35	9.15	0.007242
72	Bilijaya	2.06032	1.148888	68.345	26.525	7.135	0.004397
73	Magura-s	0.177363	0.147212	54.73	23.785	12.275	0.003961
74	Kaniar	0.901128	0.186343	85.095	21.935	12.67	0.005656
75	Balisaralaktimachi-k	0.21553	0.123961	58.15	23.38	9.49	0.004041
76	Landi	1.516675	0.318069	89.285	22.4	10.79	0.005713
77	Chinamal	0.641115	0.074242	68.775	25.415	8.785	0.004397
78	Sreyas	0.329528	0.225958	84.45	23.55	14.08	0.003731
79	Pk-21	0.481659	0.316154	86.57	21.88	15.955	0.006153
80	Jaya	0.164764	0.112979	58.2	35.04	12.43	0.003784
81	Cheruvirippu	0.462642	0.30488	86.655	22.765	12.805	0.004164
82	Sugandha-2	0.772952	0.588263	71.16	23.775	9.435	0.004867
83	Uttarbanga local-9	0.234177	0.261186	59.185	26.045	9.595	0.003274
84	Palinadhan-1	0.297542	0.306401	71.53	25.45	7.865	0.004607
85	BodiKaberi	1.40859	0.364863	83.255	20.77	9.855	0.007538
86	Barda	1.509921	0.455527	67.9	25.02	7.73	0.006193
87	Kaberi	1.395857	0.37643	64.255	25.235	9.31	0.005983
88	Kakudi manji	1.402308	0.342022	59.995	25.79	6.68	0.005073
89	Phourrel	4.93065	3.240637	60.095	24.72	11.78	0.00468
90	Kakchengphou	0.481322	0.430654	63.15	24.555	13.11	0.00452
91	Chingphou	3.072823	2.330328	19.2	21.12	11.81	0.004674
92	Phoaujaarangbele	2.81328	2.057926	66.975	24.48	9.115	0.004727
93	Turnaianganba	1.229076	0.950451	65.975	25.33	8.785	0.006342
94	Memabalbok	0.74819	0.370947	64.05	25.49	11.705	0.003857
95	Mimagisim	0.373944	0.236999	55.75	24.925	10.265	0.006939
96	Mimahambel	1.013983	0.67764	67.98	19.615	11.4	0.006546
97	Latachaunri	0.520196	0.166953	93.285	20.47	10.595	0.005976
98	Champaeisiali	0.437713	0.144814	91.775	21.265	10.305	0.006176
99	Kathidhan	0.056175	0.331524	86.165	23.34	9.77	0.00488
100	Kapanthi	0.723497	0.130731	39.6	45.175	5.56	0.005936
101	Kantakapura	1.034579	0.150765	95.28	21.235	10.585	0.006296
102	Ezhoml-2	0.544923	0.395719	87.805	22.255	13.995	0.004837
103	Kozhivalan	0.811455	0.450512	84.325	23.045	14.505	0.005743
104	PK6	0.392893	0.271173	83.715	23.505	15.16	0.004237
105	Adira-2	1.039517	0.631606	83.13	21.515	14.21	0.006163
106	Joha	0.177296	0.170112	64.09	22.275	10.65	0.003831
107	Basumati-B	0.139229	0.159013	68.18	24.405	9.605	0.0053
108	Dadghani	1.007633	0.677699	65.34	24.76	10.695	0.004677
109	Baranga	1.47834	0.39857	90.85	20.73	12.425	0.005503
110	Radhabati	1.60497	0.52335	69.795	24.27	9.835	0.006709
111	Tikichudi	2.296111	0.860473	87.475	21.515	12.375	0.005423
112	Lalmunduria	1.769935	0.567629	81.465	21.74	12.46	0.00493
113	Changli	2.192258	1.628559	64.155	25.08	11.495	0.00503
114	Mayangkhang-II	1.15973	0.779345	59.3	24.43	11.4	0.00432
115	Moiranghouanganba	2.409469	1.18002	63.94	24.6	9.58	0.004051
116	Kabokphou	1.292744	0.892616	60.305	23.705	11.3	0.004177
117	Manipurlocal	0.532425	0.327136	62.475	24.145	9.965	0.004374
118	ManoharSali	0.399378	0.225315	69.145	25.605	8.065	0.004873
119	Bengalijoha	0.367628	0.225607	63.92	25.8	11.285	0.004564
120	Anapachidhan	0.760655	0.45098	64.725	25.26	7.575	0.006466
	Mean	1.1923	0.6810	73.8762	23.6727	10.4328	0.0053
	CV%	6.5	9.3	2.4	2.3	3.9	10.2
	LSD5%	0.154126	0.139249	2.74048	1.296716	0.98272	1.27E-03

*Chl. a* Chlorophyll a content, *Chl. b* Chlorophyll b content, *Starch* Starch content, *Amylose* Amylose content, *TP* Total protein content, *TSS* Total soluble sugars content

### Genotype-by-trait biplot and correlation analyses

**Fig. 3:**
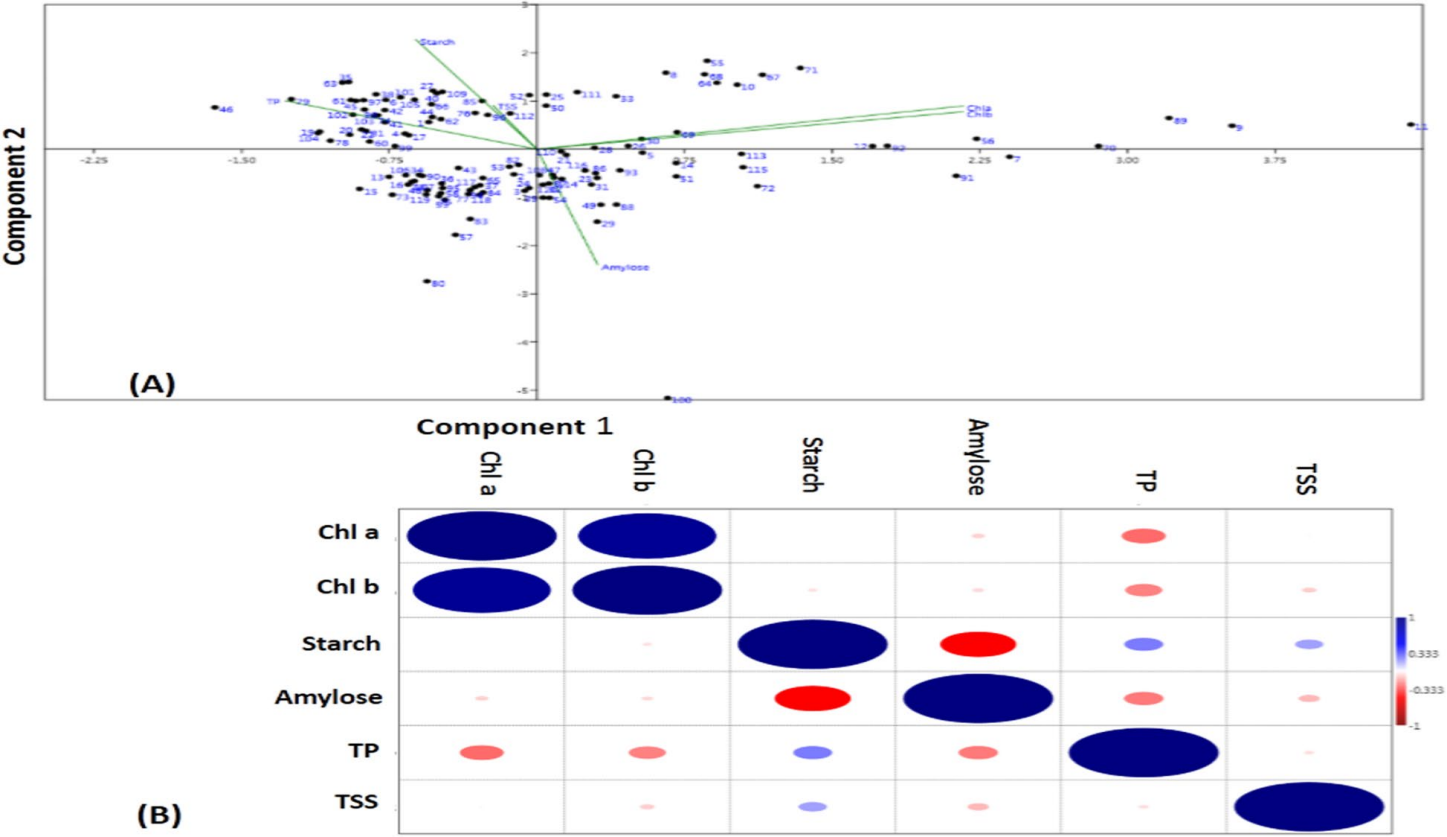
**A**. Bi-plot diagram drawn in two principal components for chlorophyll a, chlorophyll b, starch, amylose, total protein and total soluble sugars traits estimated from the 120 rice landraces. Table 1 contains the dot numbers depicted in the diagram for the serial number of the germplasm line used in the diagram and **B**. Heat map depicting Pearson’s correlation coefficients for the 6 biochemical traits. Significant correlations are color either in blue hues (positive correlation at 0.01 level) or red (negative correlation at 0.01 level)

The first two principal components were used to plot the scatter diagram for the 6 biochemical traits in the panel germplasm population of 120 genotypes and genotype-by-trait biplot graph was generated (Fig. 3A). The first and second principal components recorded 34.6 and 28.24 of the total variability with eigen values of 2.079 and 1.694, respectively. Among the 6 biochemical traits, Chl. a showed maximum diversity followed by Chl. b and total protein content based on the principal component analysis of the panel population (Fig. 3A). The PCA diagram distributed the germplasm lines in all the 4 quadrants based on the 6 traits in the genotypes. All the high protein containing germplasm lines were in the quadrant IV (top left). All the high chlorophyll carrying germplasm lines were placed in the quadrants I (top right) and II (bottom right). The genotypes containing high estimates for all the six traits were not seen in any particular germplasm line. Thus, for selection of donor parents for the six traits, we need to select at least 2 germplasm lines as parental line for the improvement of these six traits.

The correlation analysis in the panel population revealed that chlorophyll had a strong positive correlation with chlrophyll b content. A strong positive correlation was observed between the starch and amylose content. In addition, total protein content and total soluble sugars content also showed strong positive correlation in the mapping population. A negative correlation was recorded for starch content with amylose content. In addition, total protein content showed negative correlation with amylose content. A negative association is also observed for chlorophyll content with total protein content (Fig. 3B).

### Cluster analysis

**Fig. 4:**
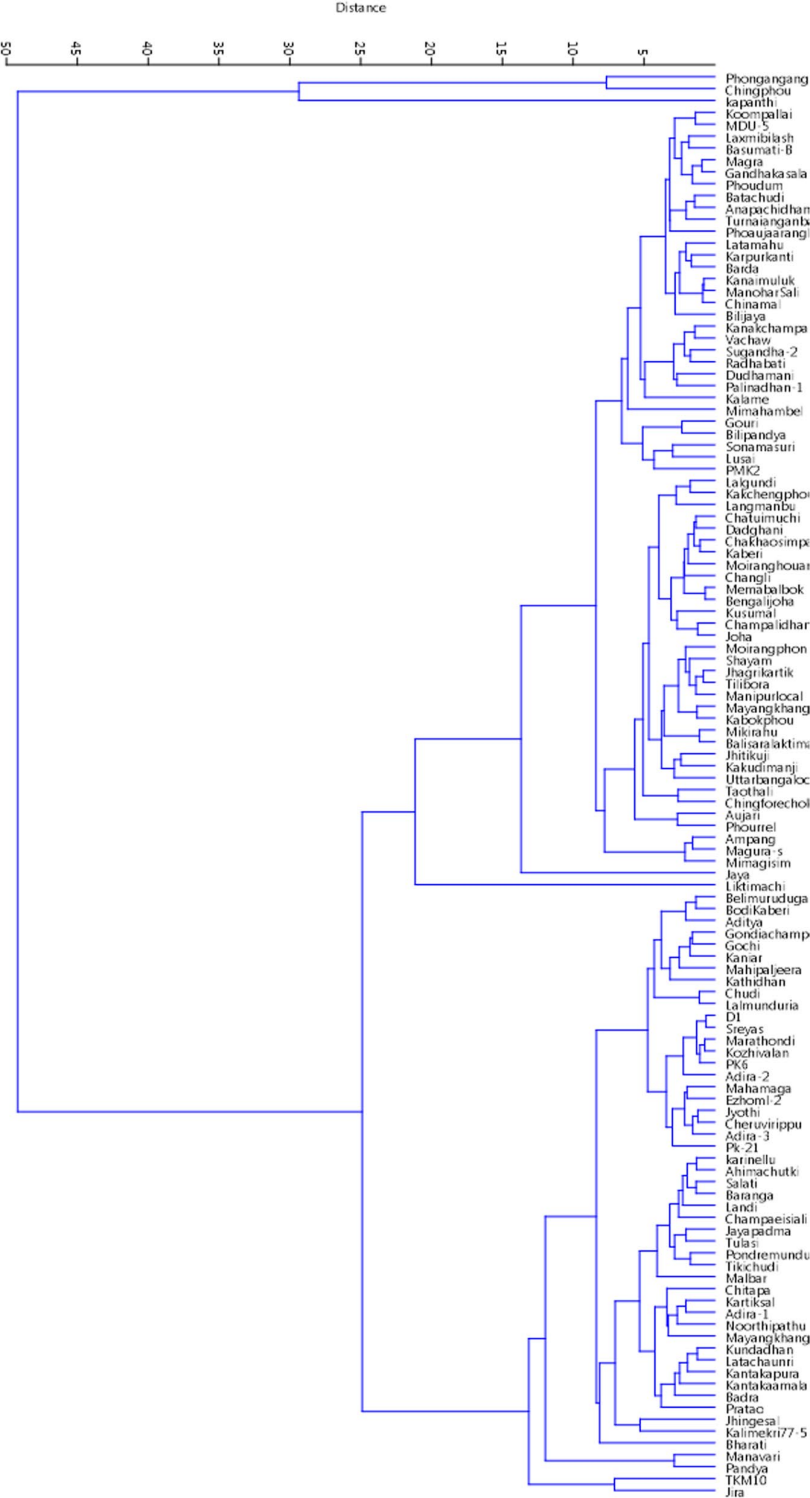
Wards’s clustering approach based on the estimates of 6 biochemical traits for clustering of 120 germplasm lines

Panel containing 120 genotypes were broadly clustered into two groups based on the mean values of the six studied biochemical traits. The smaller cluster accommodated 3 genotypes together as they showed low values for TSS, Chl. a, Chl. b, starch and amylose content. The bigger cluster consists of rest of the 117 genotypes. This cluster was again divided into two sub-clusters, one having 66 and other with 51 genotypes (Fig. 4). The sub-cluster I included 66 genotypes were grouped together having medium to low and very low mean values for starch content and medium to high and very high values for amylose content. The other sub-cluster II with 51 genotypes was grouped for high to very high starch and amylose content.

the sub-cluster I was grouped into two based on starch content, where only one genotype, Liktimachi included with very low starch content and rest 65 genotypes in the other, where the starch content ranges from low to very high. Amylose content had divided the sub-cluster I with 65 genotypes again into two groups with Jaya having very high amylose content form one group and rest 64 genotypes with mean values for amylose content ranging from medium to high only grouped into second one. The group with 64 genotypes were again assembled to give two sub-clusters having 31 (all having similar, i.e. medium values for starch content) and 33 (starch content ranging from medium to low and amylose content ranging from medium to high) genotypes. The sub-cluster II with 51 genotypes were sub-grouped into two: one with TKM10 and Jira, both with high mean values for amylose and starch content and very low values for TP; and other having 49 genotypes showing similarity for starch and amylose content ranging from low to high mean values.

The sub group with 49 genotypes was again grouped into two, as per similarities of starch, amylose and TP. This gave rise to two groups, one including Manavari and Pandya and other with the rest 47 genotypes. Manavari and Pandya were similar, both having very high- starch and medium values for amylose and TP content. The mass with 47 genotypes ranged high to very high for starch, low to medium for amylose and low – very high for TP values. This group was divided into sub-groups with 22 and 25 genotypes. The cluster with 22 genotypes was similar at a point having similarities for mean values: Chl. b medium to very low, starch- high, amylose- medium and TP- medium to very high. The other one with 25 genotypes were found to have mean values ranging from high to very high for starch and low to medium for amylose.

### Assessment of molecular diversity using the SSR markers

Diversity of the panel population was assessed using the estimated diversity parameters by genotyping the population using 136 SSR markers. A total of 506 markers alleles were detected from the population which indicated that the population is diverse (Supplementary Table 3). Also, the allele frequency varied from 2 to 7 alleles per marker with an average value of 3.72/marker. Highest numbers of alleles were produced by the marker RM493. This indicated that the markers were effective in characterizing the panel population. The specific allele frequency was observed to be highest (0.916) in the germplasm line TKM10 detected by marker RM22034. The average frequency detected for specific allele of the population was high (0.561).

The panel population showed maximum gene diversity by the marker, RM493 with a value of 0.813 while a low diversity value of 0.142 was detected by the marker, RM6054. The average gene diversity value in the population was 0.5545. A total of 29 markers viz., RM328, RM1812, RM6947, RM4978, RM22034, RM258, RM1347, RM315, RM3423, RM405, RM421, RM317, RM502, RM6641, RM282, RM11701, RM112, RM509, RM16686, RM6091, RM209, RM245, RM3351, RM471, RM467, RM8007, RM518, RM274, and RM452 showed nil allele heterozygosity in the population. The maximum value of allele heterozygosity was 0.958 showed by the marker, RM3735. The mean heterozygosity in the population was 0.114 detected by 136 SSR markers. The polymorphism information content value for measurement of the informativeness of genetic markers showed highest value of 0.787 by the markers, RM493. The PIC mean value of 136 markers was estimated to be 0.496 in the population.

### Genetic structure analysis

**Fig. 5:**
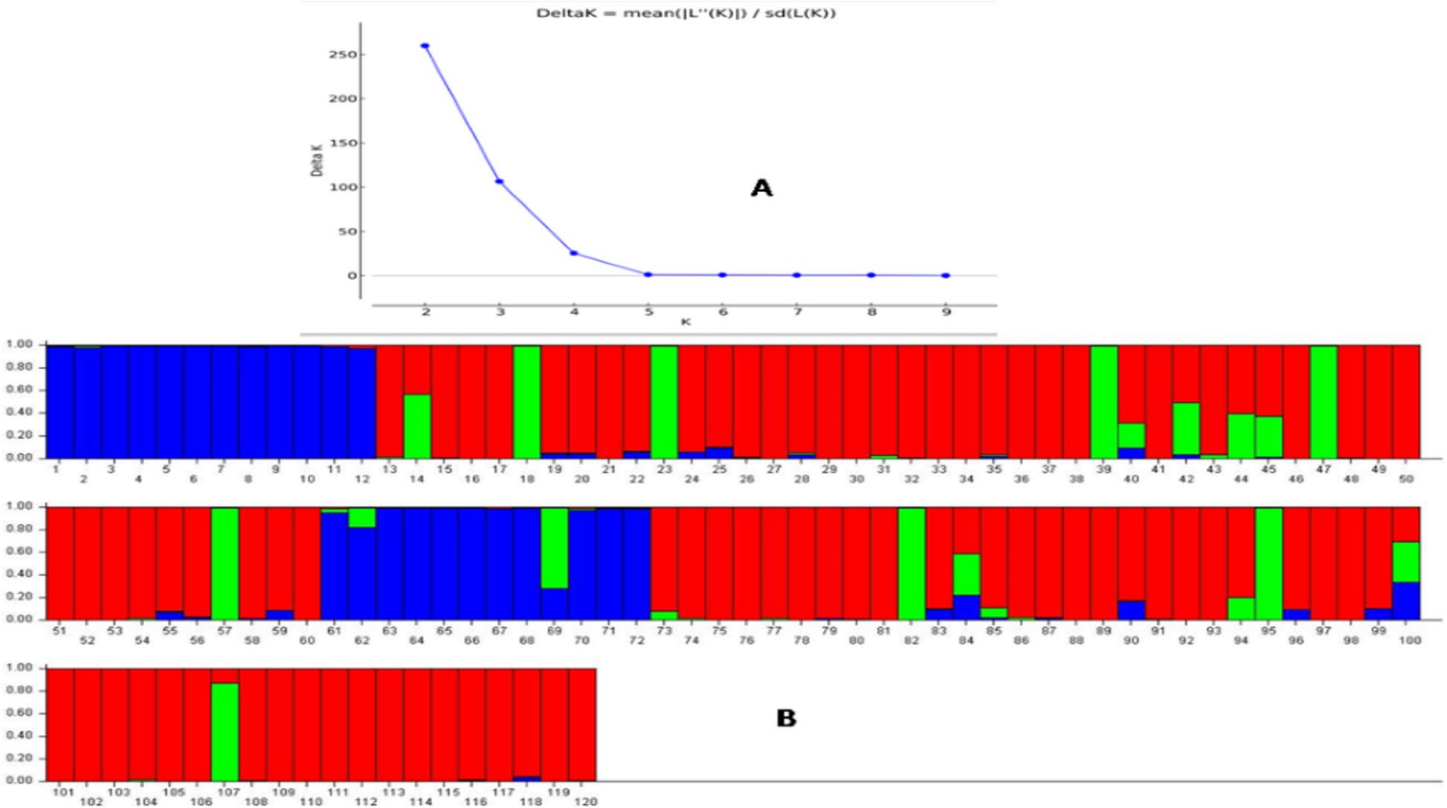
**A**. Plot of ∆K value to the K value for the rate of change in the log probability of data between successive K values and **B**. Population genetic structure for the panel population based on the membership probability fractions of individual members at K = 3. The member with the inferred probability of ≥80% membership proportions were taken as subgroups while others classified as admixture line. Table 1 contains the serial number of the germplasm lines depicted in the diagram

The population genetic structure analyzed by the STRUCTURE software grouped the panel population into subgroups based on the peak ∆K value at an assumed K value. The highest peak of ∆K value (259.77) obtained at K = 2 and the whole population was divided into two subpopulations (Supplementary Fig. 1). However, the two subpopulations produced did not correspond well with the six biochemical traits estimated from the panel. Therefore, next ∆K peak value (106.54) at K = 3 was considered for classification of the panel population. The three sub populations obtained based on the ∆K peak that is by genotyping of 136 SSR markers (Fig. 5). The sub populations obtained at K = 3 showed a good correspondence with each of the studied biochemical traits (Supplemental Table 4). The genotypes with ≥80% probability were assigned to the corresponding subpopulation and the rest as admix genotypes. The sub-population 1 accommodated 81 genotypes of which majority were poor and very poor for the target traits. The sub-population 2 included 8 germplasm lines of which majority were with moderate in content of target traits. The sub-population 3 which accommodated 23 genotypes were for the majority of the high and very high carrying target traits while the rest germplasm lines were admix genotypes. The inferred cluster distances for the proportion of the germplasm lines were 0.689, 0.102 and 0.208 in sub-population 1, sub-population 2 and sub-population 3, respectively. The three subpopulations showed fixation indices (Fst) values of 0.1641, 0.375 and 0.3418 for sub-population 1, sub-population 2 and sub-population 3, respectively.

The net nucleotide distance (allele-frequency divergence) of sub-population 1 and sub-population 2 was 0.1704; sub-population 1 and sub-population 3 estimated to be 0.1186 while between 2 and 3 sub-populations was estimated to be 0.2302. The average distance (expected heterozygosity) among the members in sub-population 1 was 0.4264; within the individuals in sub-population 2 was 0.3901 while 0.3783 was computed for sub-population 3. The population structure analysis classified the population into sub-populations based on thepeak value of ∆K at K = 3 (Fig. 5). Majority of the germplasm lines containing high and very high estimates of the biochemical traits were found in the subpopulation 3 (SP 3; Blue color) while moderate value carrying germplasm lines were in the subpopulation 2 (SP 2; Green color). The germplasm lines with low and very low in the six biochemical traits were found in the sub-population 1 (SP 1; Red color). The alpha value estimated by the structure software at K = 3 for the panel population was very low (alpha = 0.046). The alpha-value showed a leptokurtic distribution for the panel population while the Fst values of each sub-population were distributed almost symmetrically at K = 3 (Supplementary Fig. 2).

**Fig. 6:**
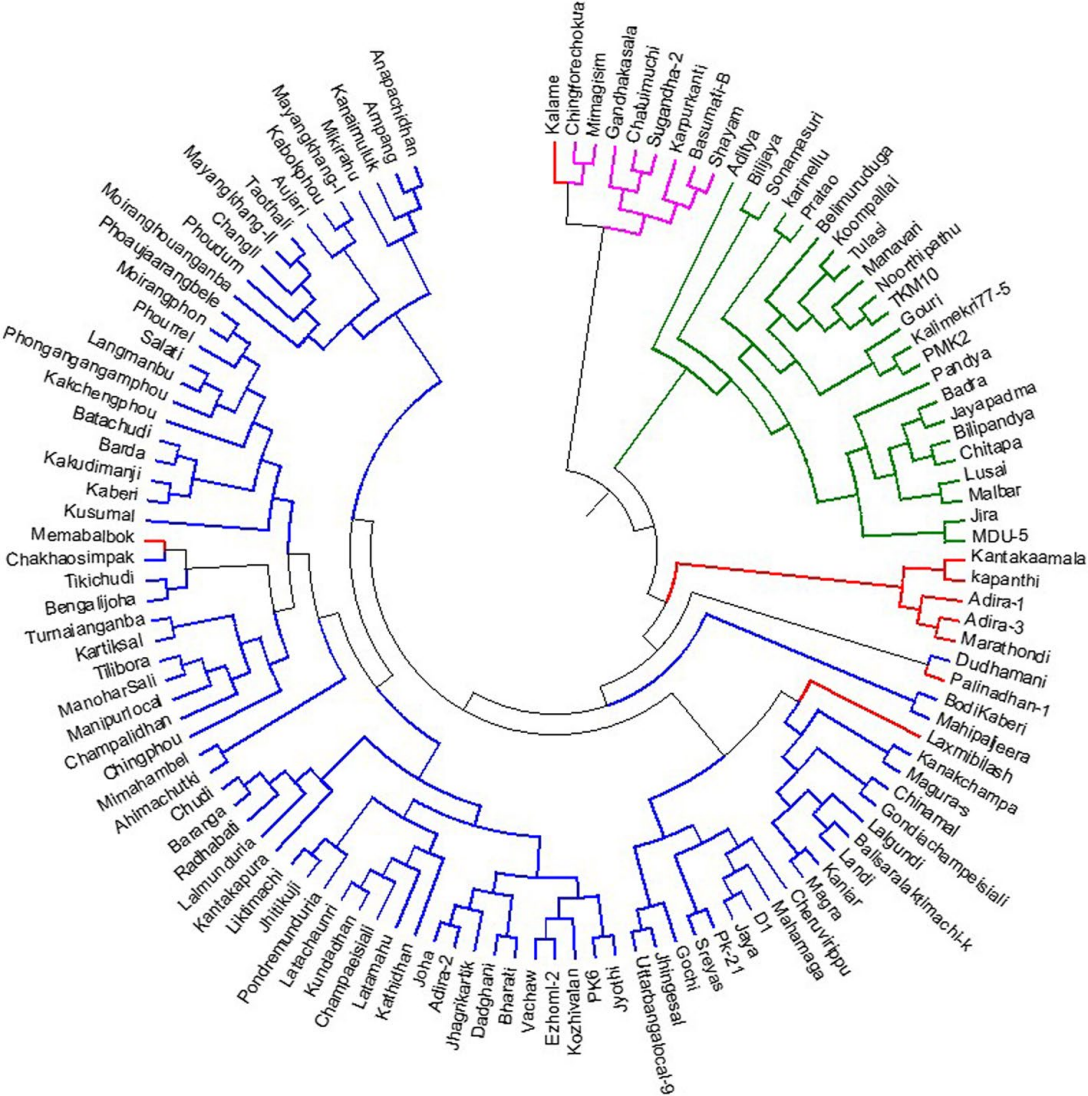
UPGMA un-rooted tree constructed based on the genotyping results of 120 germplasm lines using 136 SSR markers for the clustering of the sub-populations obtained from structure analysis at K = 3 (SP1: Blue; SP2: pink; SP3: green and Admix: brick red)

The cluster analysis grouped the genotypes on the basis of genotyping results using 136 SSR markers data and placed the germplasm lines into different clusters which showed correspondence with the studied biochemical traits in the germplasm lines. The UPGMA tree differentiated the genotypes into traits in the 4 different clusters (Fig. 6). The clusters accommodated various germplasm lines as per the structure sub-population and majority of the germplasm lines were in sub-population 1 depicted in blue color in the tree (Fig. 6). The admix type germplasm lines of the population are depicted in brick red color in the neighbour joining tree while the members of the subpopulation 2 are in pink color (Fig. 6).

### Molecular variance (AMOVA) and LD decay plot analysis

The members present in a sub-population show similarity among themselves for various traits of the population. The analysis of molecular variance (AMOVA) was performed in a population to know the genetic variations present within and between the sub-populations (Table 2). The genetic variations estimated considering the K value at K = 3 and computed to be 8% among the populations, no variation among individuals and 92% within the individuals of the panel population. The deviation from Hardy-Weinberg’s prediction was checked from the estimates of Wright’s F statistics. The uniformity of individuals within a sub-population was checked using the F_IS_ parameter estimated for the differentiation of the sub-populations while the statistics, F_IT_ was used to know the variation of individual within the total population for the differentiation in a population. The estimates of F_IT_ and F_IS_ of the total population and within population were − 0.148 and − 0.235 based on the genotyping of 136 marker loci. The total population showed mean F_ST_ value of 0.071 for the 4 sub-populations. The subpopulations or population differentiation was estimated on the basis of Fst values of each subpopulation in a population. The Fst values of each of the 4 subpopulations clearly differentiated the subpopulations based on their values and distributions pattern (Supplemental Fig. 2).

**Table 2:** Analysis of molecular variance (AMOVA) of the sub-populations present in the panel population containing 120 germplasm lines

Sources of variation	AMOVA for the four sub-populations at K = 3			
	df.	Mean sum of squares	Variance components	Percentage variation
Among populations	3	177.669	3.726	8
Among individuals (accessions) within population	116	26.016	0.000	0.0
Within individuals (accessions)	120	41.842	41.842	92
Total	239	8571.825	45.568	100
F-Statistics	Value	*P*-value		
F_ST_	0.099	0.001		
F_IS_	−0.233	1.000		
F_IT_	−0.111	1.000		
F_ST_ max.	0.502			
F’_ST_	0.197			

**Fig. 7:**
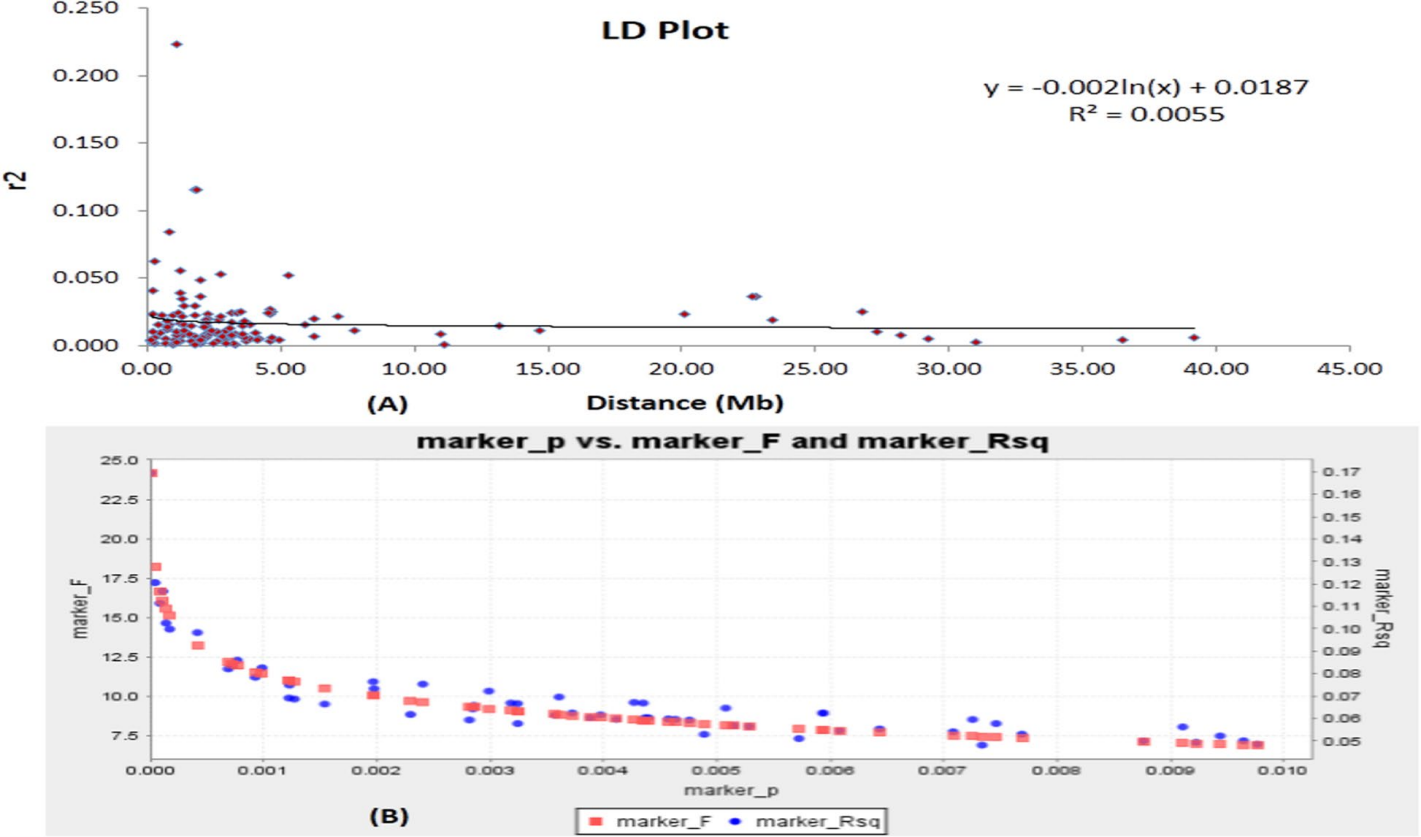
**A**. The physical distance (Mb) between pairs of loci on chromosomes against linkage disequilibrium (LD) decay (*r*^2^) curve plotted in rice; **B**. The marker ‘P’ versus marker ‘F’ and marker *r*^*2*^ detected. The decay started in million bp estimated by taking 95^th^ percentile of the distribution of *r*^*2*^ for all unlinked loci

The association of alleles is dependent on the existence of traits in LD in a population for utilization of marker-trait association. Continuance of marker–trait association in a populationis dependent on the LD decay rate over a time period. The existence of different inferred value in a germplasm line may depend on the LD decay rate in a population. New admix type will indicate the possibility of new genes or allelic variants for the target traits in a population. The LD plot was constructed using the syntenic *r*^2^ value in a population versus the markers physical distance in million base pair to know the trend of linkage disequilibrium decay in the population (Fig. 7A). The tightly linked markers showed higher *r*^2^ value and the average *r*^2^ values decreased rapidly for the increase in linkage distance. The LD plot revealed that the decay was delayed in the beginning in the panel population for the studied traits. The LD decay was declined for the associated markers in the curve at about 1-2 M base pair and thereafter a very slow and gradual decrease was noticed from the plot. It clearly revealed the continuance of linkage disequilibrium decay in the population for the studied six biochemical traits. The estimate of LD decay may be influenced under the situation of mutation, non-random mating, selection, migration or admixture, and genetic drift. The clue for creation of genetic admixture groups in the population for various biochemical traits is indicated from the LD decay plot. The plot of marker ‘P’ versus marker ‘F’ and marker *r*^*2*^ also showed a similar trend in the curve (Fig. 7B). The associated markers detected from this analysis provided the strength of the markers for use in the improvement programs of biochemical traits.

### Principal coordinates and cluster analyses for genetic relatedness among the germplasm lines

**Fig. 8:**
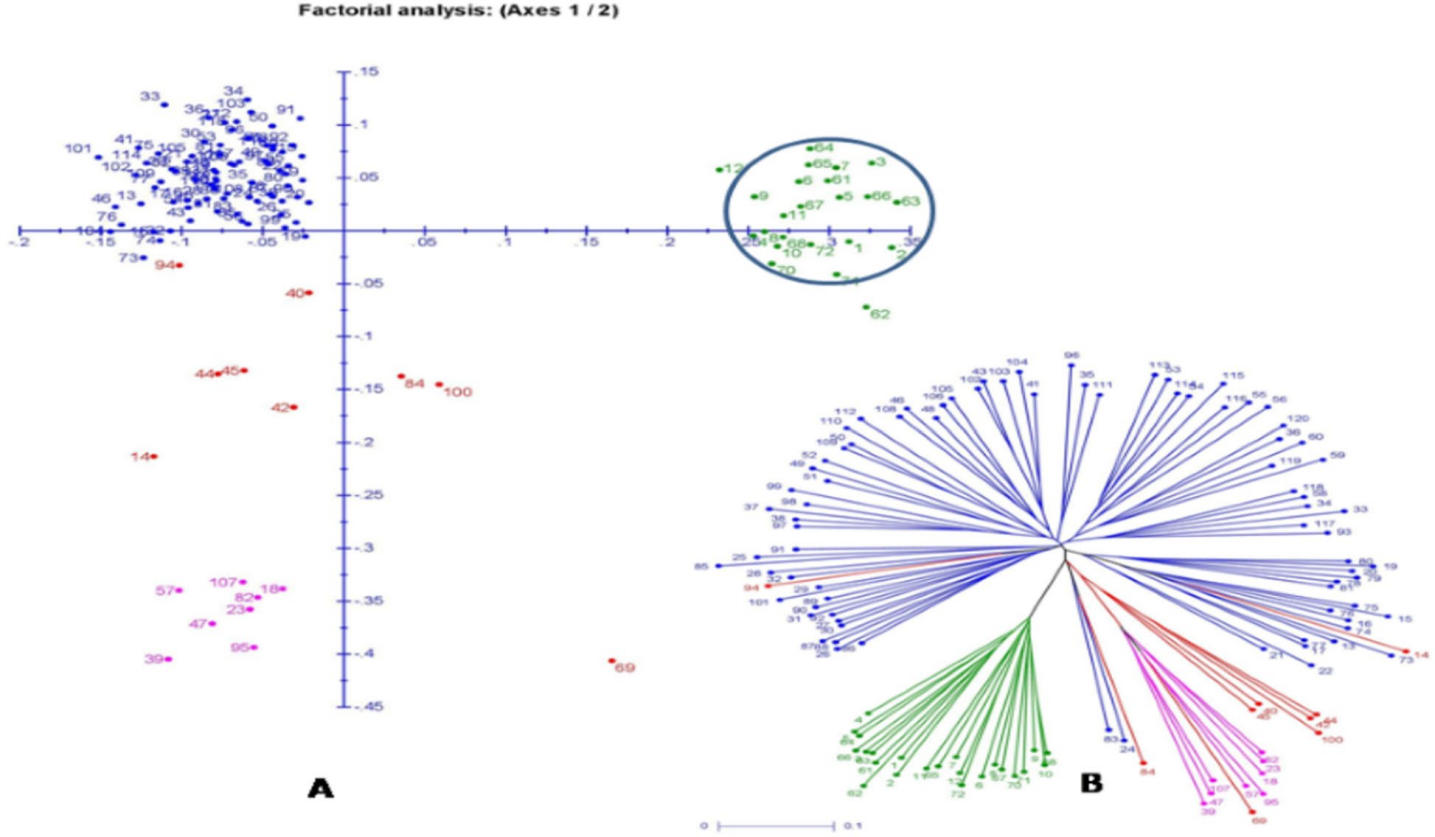
**A**. Distribution of the 120 landraces present in the panel population for 6 biochemical traits using 136 molecular markers in the principal coordinate analysis (PCoA) plot, **B**. Depicts the neighbour-joining tree color based on the sub-populations from structure analysis at K = 3. The serial number of the genotypes depicted as dot numbers in the tree as in the Table 1. The colors are SP1: blue; SP2: pink; SP3: green and Admix: brick red on the basis of sub-populations obtained from structure analysis

The principal coordinate analysis (PCoA) in the two dimensional plot was constructed based on the marker data of genotyping results using 136 SSR markers that grouped the 120 panel germplasm lines on their genetic relatedness among the members (Fig. 8A). The inertia for component 1 was 11.59% while component 2 showed 7.49%. The genotypes were grouped in the four different quadrants making 2 major and 2 minor groups (Fig. 8A). The biggest group accommodated almost all the germplasm lines of the subpopulation 1 carrying low quantity of biochemical traits and depicted in blue color. The quadrant I and II formed a group and accommodated majority of sub-population 3 carrying high estimates of the biochemical traits. The members of the sub-population 2 were present in the quadrant III (bottom left) in pink color. The admix types are present in the quadrant II and III and depicted in brick red color (Fig. 8A).

The un-rooted tree is reared using phylogenetic tree. The tree indicates no common ancestor or node in the tree. The germplasm lines containing high to very high estimates of biochemical traits are grouped together forming the sub-population 3. This group is depicted in green color in the un-rooted tree (Fig. 8B). The variations can easily be assessed among the landraces from the distance of each landrace depicted in the tree (Fig. 8B). The relationship is estimated in both the trees here without considering the evolutionary time of the landraces.

### Marker-trait associations with biochemical traits in rice

**Fig. 9:**
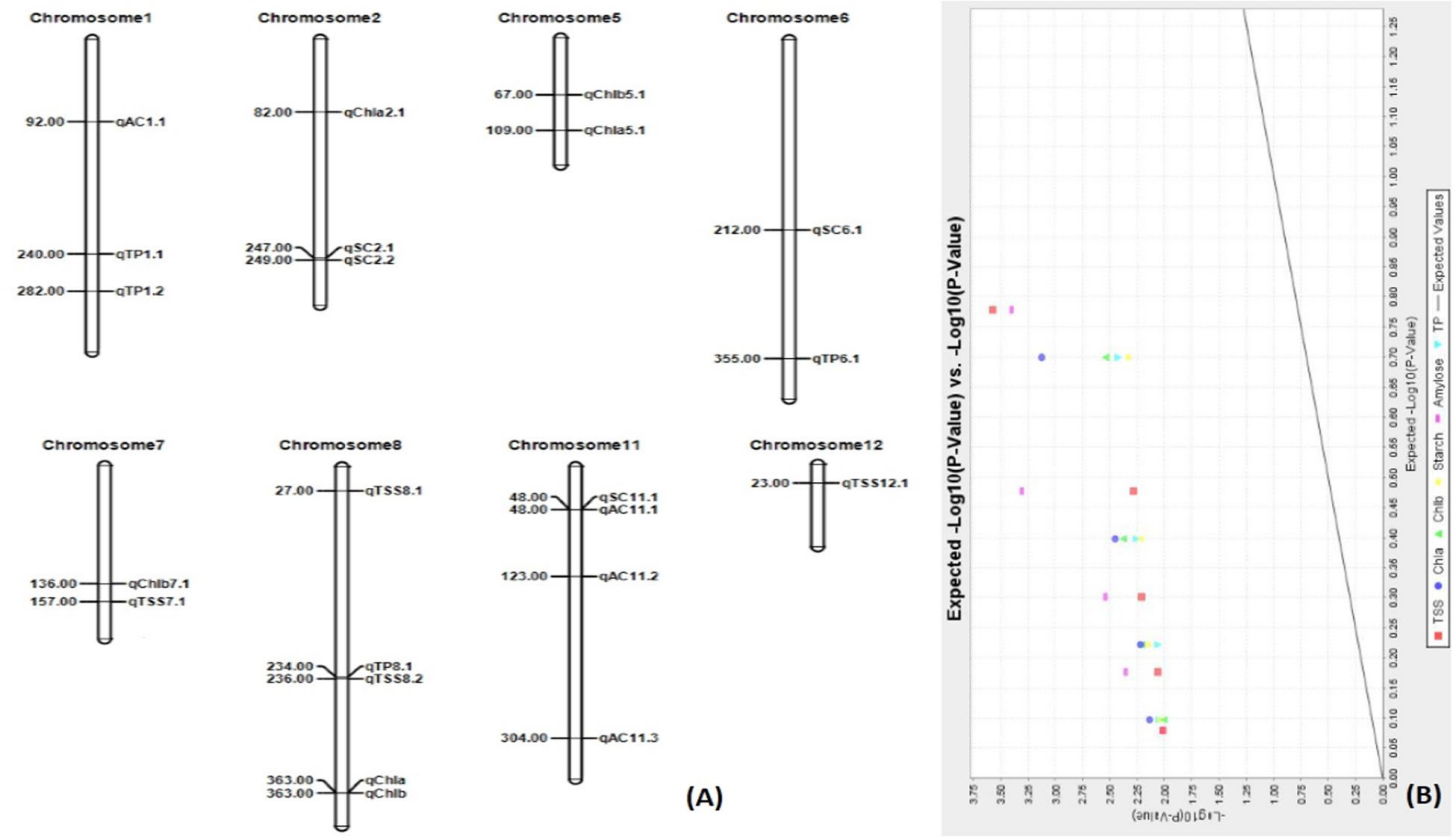
**A**. The positions of the QTL on the chromosomes for chlorophyll a, chlorophyll b, starch, amylose, total protein and total soluble sugars **B**. Distribution of marker-trait association and quantile–quantile (Q-Q) plot generated from Mixed Linear Model analysis for the six biochemical traits detected by association mapping at *p* < 0.01 in rice

Association of six biochemical traits with molecular marker was performed using TASSEL 5 software adopting the GLM and MLM approaches. The associations were detected at both < 1 and < 5% error. The six traits viz.*,* total protein content, total soluble sugars, starch, amylose, chlorophyll a and chlorophyll b content were detected to be above the threshold level and found to be associated with the SSR markers using the GLM and MLM approaches (Table 3). While analyzing by model GLM at 5% level, 200 markers-traits associations were observed. But, 60 markers-traits associations were detected by GLM analysed at < 1% error (Supplementary Table 5). The analysis by MLM approach showed 110 associations at < 5% error while 26 associations were detected at < 1% level (Supplementary Table 6). However, while considering both GLM and MLM approaches at < 1% error level, 21 significant marker-trait associations were detected. Three significant marker-trait associations for each of Chl. a, Chl. b and starch content were detected while 4 associations were computed for TSS, TP, amylose content by both the models (Table 3; Fig. 9A). The markers detected by association study by both GLM and MLM approaches are considered as robust markers. The generated Q-Q plot also confirmed the association of the markers with 6 biochemical traits in rice (Fig. 9B).

**Table 3:** Marker-trait association for chlorophyll a, chlorophyll b, starch, amylose, total protein and total soluble sugars in rice landraces present in the panel population detected by both the models of GLM and MLM analyses at *p* < 0.01

Sl. No.	Traits	Markers	Chr#	Positions (cM)	GLM				MLM			
					Marker_*F*	Marker_*p*	*q* value	Marker_*r*^*2*^	Marker_*F*	Marker_*p*	*q* value	Marker_*r*^*2*^
1	Chl. a	RM1347	2	82	12.06469	7.23E-04	0.00461	0.08366	8.85234	0.00356	0.00953	0.07251
2	Chl. a	RM405	5	109	18.22659	4.03E-05	0.00121	0.12059	7.46088	0.00729	0.00998	0.06111
3	Chl. a	RM3231	8	363	16.6411	8.32E-05	0.00163	0.11142	11.98915	7.50E-04	0.00488	0.0982
4	Chl. b	RM440	5	67	11.55526	9.27E-04	0.00493	0.07842	7.76369	0.00623	0.00953	0.0628
5	Chl. b	RM5436	7	136	7.80936	0.00608	0.00760	0.0546	6.86045	0.00999	0.00999	0.05549
6	Chl. b	RM3231	8	363	15.11793	1.69E-04	0.00169	0.09981	9.27211	0.00288	0.00953	0.075
7	Starch	RM3701	11	48	9.07463	0.00318	0.00712	0.06686	7.79603	0.00613	0.00953	0.06645
8	Starch	RM20377	6	212	10.02303	0.00198	0.00660	0.07329	8.32763	0.00466	0.00953	0.07099
9	Starch	RM6374	2	249	11.93771	7.69E-04	0.00461	0.08598	7.03173	0.00913	0.00999	0.05994
10	Amylose	RM3701	11	48	13.21964	4.14E-04	0.00355	0.09824	13.32049	3.95E-04	0.00426	0.11376
11	Amylose	RM315	1	92	7.41586	0.00746	0.00845	0.0577	8.42245	0.00444	0.00953	0.07193
12	Amylose	RM167	11	123	10.03053	0.00197	0.00660	0.07642	9.27197	0.00288	0.00953	0.07918
13	Amylose	RM6091	11	304	16.06201	1.09E-04	0.00163	0.11679	12.86845	4.91E-04	0.00426	0.1099
14	TP	RM566	8	234	8.23343	0.00489	0.00716	0.05289	7.10636	0.00878	0.00999	0.05832
15	TP	RM220	1	240	10.52602	0.00154	0.00578	0.06639	7.08199	0.00889	0.00999	0.05812
16	TP	RM5638	1	282	10.91588	0.00127	0.00508	0.06864	8.73341	0.00378	0.00953	0.07168
17	TP	RM253	6	355	9.72444	0.00229	0.00712	0.06172	7.99151	0.00554	0.00953	0.06559
18	TSS	RM247	12	23	8.46122	0.00435	0.00712	0.06682	7.12111	0.00871	0.00999	0.06081
19	TSS	RM337	8	27	7.46984	0.00726	0.00845	0.05946	8.09955	0.00524	0.00953	0.06916
20	TSS	RM248	7	157	24.15956	2.94E-06	0.00018	0.16942	14.13975	2.67E-04	0.00426	0.12074
21	TSS	RM566	8	236	8.82874	0.00361	0.00712	0.06952	7.78093	0.00617	0.00953	0.06644

*Chl. a* Chlorophyll a content, *Chl. b* Chlorophyll b content, *Starch* Starch content, *Amylose* Amylose content, *TP* Total protein content, *TSS* Total soluble sugars content

Chlorophyll a and Chlorophyll b content showed significant association with 3 markers each analyzed by both the models. The associations of the SSR markers RM1347, RM405 and RM3231 with Chl. a are located on chromosome 2, 5 and 8 at 82, 109 and 363 cM positions, respectively. The trait, Chl. b showed significant association with the markers RM440, RM5436 and RM3231 (Table 3). Th**e** starch content showed association with the markers RM3701, RM20377 and RM6374. Amylose content showed association with the markers, RM3701, RM315, RM167 and RM6091 analyzed by TASSEL using both the models. Four markers namely RM556, RM220, RM5638 and RM253 showed associations with protein content estimated from the panel population. RM 220 is located on Chromosome 1 at 4.4 Mb position showing about 0.06 marker *r*^*2*^ value detected by both the models. RM 5638 is also present on chromosome 1 at 20.9 Mb position with about 0.07 marker *r*^*2*^ value. The marker, RM253 is present on chromosome 6 at 5.4 Mb position showing marker *r*^*2*^ value of > 0.06 by both the models. RM 556 is present on chromosome 8 at 22.3 Mb position with > 0.05 *r*^*2*^ value. The total soluble sugars present in the panel germplasm lines showed associations with markers RM247, RM337, RM248 and RM566 analyzed using both models of GLM and MLM.

The QTL for Chl. a and Chl. b on chromosome 8 at 363 cM position are detected to be co-localized showed association with the marker, RM3231. Another two QTL on chromosome 8 at position 234 cM controlling protein and total soluble sugars content were found to be co-localized. Similarly, the traits starch and amylose content were significantly associated with marker, RM3701 and detected to be closely located on chromosome 11 at 48 cM position.

## Discussion

Protein, starch, amylose and total soluble sugars are basic metabolites of seed that influence the eating, cooking and nutritional qualities of rice. Chlorophyll is responsible for the absorption and exploitation of the light energy influencing photosynthetic efficiency in rice. The results of the study showed wide genotypic variation among the germplasm lines for protein, starch, amylose, total soluble sugars and chlorophyll content in the mapping population and hence the developed panel was effective for mapping of the target traits. The donor line in earlier publications for grain protein content containing 16.41% was reported in the germplasm line, ARC10063 [2, 42]. In this investigation, another landrace, Bharati showed protein content of 18%. This landrace will serve as a potential donor for protein improvement programs. The employed markers showed high PIC, gene diversity and specific alleles value in the panel population indicated a diverse panel population. Many earlier results also report high genetic diversity parameters in various rice populations [43–48]. The landraces studied in the present investigation were collected from the locations of five states known for rich genetic diversity in rice including the secondary centre of origin [49–52]. Hence, the panel population is effective for mapping of the six biochemical traits of rice.

The population genetic structure categorized the panel population into three sub-populations. The structure correlation and grain protein content in rice was reported earlier by Pradhan et al. [2]. But, population structure analysis for starch, amylose, total soluble sugars and chlorophyll using rice landraces are not available. However, structure correlation with phenotype in rice has been reported by many researchers [53–58]. Detection of many admix type landraces in the population revealed clue for evolution of the traits from different germplasm lines during the evolution process. This is also clear from the existence of many groups and subgroups in the population (Figs. 4 and 5).

The total protein content estimated from each germplasm lines from the panel showed significant associations by both the models with RM556, RM220, RM5638 and RM253. RM 220 is located on chromosome 1 at 240 cM position showing about 0.06 marker *r*^*2*^ value detected by both the models (Fig. 9A). The mapping results of Kinosita et al. [59] and Jang et al. [20] reported protein controlling QTL on chromosome 1 but quite away from the QTL detected in the present investigation. Hence, this detected QTL is not reported in earlier studies and designated as *qPC1.2*. RM 5638 is also present on Chromosome 1 at 20.9 Mb position with about 0.07 marker *r*^*2*^ value. The mapping results of Aluko et al. [60], Yang et al. [61] and Kinosita et al. [59] reported QTL for controlling protein content located on chromosome 1 at ~ 21–38 Mb which is closer to *qProt1* reported by Terao and Hirose [62]. The present investigation detected a protein controlling QTL in the same region. Therefore, the previously detected QTL, *qProt1* is validated in this study and will be useful in marker-assisted breeding program for protein content enhancement. The marker, RM253 is present on chromosome 6 at 5.4 Mb position showing marker *r*^*2*^ value of > 0.06 by both the models. Kinosita et al. [59] reported the QTL, *qPC6.2* in between marker interval position 5.2–9.7 Mb. In the present investigation we detected a QTL within this marker position similar to Kinosita et al. [59]. Therefore, the previously detected QTL, *qPC6.2* is validated in this study and will be useful in marker-assisted breeding program for protein content enhancement. RM556 is present on chromosome 8 at 22.3 Mb position with > 0.05 *r*^*2*^ value. The QTL reported by Yun et al. [63] was within the marker interval of 19.3–26.35 Mb on chromosome 8. The QTL reported by us is within the marker interval of reported by Yun et al. [63] is validated in this study. The QTL was not assigned any designation by Yun et al. [63] and hence the QTL is designated as *qPC8.2* (Fig. 9A).

Significant marker-trait associations for total soluble sugar were detected to be associated by the markers RM247, RM337, RM248 and RM566 through analyzing by both GLM and MLM approaches. In a mapping study by Yang et al. [61], reported QTL for the total soluble sugar, *qSS8.1* at the marker interval of RM1235-RM1376 in the region 25-30 cM position. In our study, RM337 located at 27 cM on chromosome 8 was associated strongly and controlled total soluble sugar in the population. The QTL, *qTSS8.1* reported by Yang et al. [61] is validated in this present study and will be useful for total soluble sugars improvement programs in rice. No genes or QTL were reported in previous studies for total soluble sugar detected on chromosomes 7, 8 and 12 at 157, 236 and 23 cM position. These QTL are designated as *qTSS7.1*, *qTSS8.2* and *qTSS12.1*_*,*_ respectively. The marker, RM248 showed very high marker *r*^*2*^ value of > 0.12 with total soluble sugars analyzed by both the models and present on chromosome 7 at 157 cM position. The marker-trait associations were detected by both the models (GLM and MLM) at *p* < 0.01, low *p* value, high *r*^*2*^ value (Table 3) and Q–Q plot also confirmed the associations ofthese markers (Fig. 9B). These strongly associated SSR markers RM248, RM566 and RM247 for total soluble sugars trait may be useful for marker-assisted program in improving total soluble sugars in rice.

In our investigation, the amylose content is detected to be significantly associated with the marker, RM315 on chromosome 1 at 92 cM position. Li et al. [64] reported a QTL, *qAC1.1* for amylose content on chromosome 1 but at 40 cM position. Zheng et al. [65] reported a QTL for amylose content as *qAC1.2* at 102.5 cM in the marker interval of C904-R2632. In the study of Swamy et al. [66] reported the QTL, *qac*_*1.1*_ for amylose content at position 60-90 cM in marker interval of RM243-RM582 on chromosome 1. Also, Swamy et al. [66], reported *qac1.2* and *gel-2* QTL for amylose content and gel consistency in marker interval of RM580–RM81 at the position 90-100 cM on chromosome 1. Thus, these reports validated for the QTL *qAC1.2* for amylose content on chromosome 1. The QTL, *qAC11.1* has been detected on chromosome 11 for amylose content at 27 cM with RM6091 showing *r*^*2*^ value 0.1099. This QTL is reported in earlier findings of Lee et al. [14] which is validated in this study. The QTL detected using both the models for amylose content on chromosome 11 at 123 cM and 304 cM by the associated markers RM167 and RM6091, respectively. No reports are available fordetection of these two QTL controlling amylose content at positions 48 cM, 123 cM and 304 cM on chromosome 11. Hence, the three QTL may be novel QTL and designated as *qAC11.1, qAC11.2* and *qAC11.3* (Fig. 9A)_*.*_

In this investigation, marker RM6374 was associated with the trait, seed starch content showing *r*^*2*^ value of 0.06383 on chromosome 2 at 249 cM. Panahabadi et al. [22] reported *qSTh2.1* for starch content on the same chromosome but at a position of 4.04 cM. This confirms *qSC2.1*at 247 cM and *qSC2.2* at 249 cM as novel QTL for seed starch content. Two QTL detected on chromosome 6 and 11 at 212 and 48 cM position were detected for starch content by analyzing in both the models (Table 3; Fig. 9A). No previous reports are available for starch controlling QTL at these positions. These two QTL may be novel and designated as *qSC6.1* and *qSC11.1*_*.*_ Yang et al. [14] reported *ALK* gene as starch synthesis gene at 12.9 cM on chromosome 6 with marker RM8200.

Chlorophyll a content is significantly associated with the SSR markers RM1347, RM405 and RM3231 located on chromosome 2, 5 and 8 at 82, 109 and 363 cM positions, respectively. The QTL, *qCH2* for Chlorophyll a on chromosome 2 was reported earlier by Kun et al. [67] within marker interval of RM327-RM123 at 80-95 cM. We also detected the QTL at 82 cM position. Therefore, the QTL, *qCH2* is validated in this mapping study and will be useful in chlorophyll improvement program in rice. However, no QTL for chlorophyll content was reported on chromosome 8 at 363 cM position. This detected QTL may be a new QTL and designated as *qChla8.1*. The QTL detected on chromosome 5 was located at 109 cM position. Ye et al. [17] reported a QTL for chlorophyll content in the interval of 110.46-118.71 cM region on the chromosome 5. The detected QTL may be the same QTL, *qSLCHH* reported by Ye et al. [17]. The trait, chlorophyll b showed significant association with the markers RM440, RM5436 and RM3231 located on chromosomes 5, 7 and 8 at 67, 136 and 363 cM positions, respectively. Zhang [68] reported a QTL, *qChlb 5D* controlling chlorophyll on chromosome 5 at 68.2 cM position. The QTL detected by us at 67 cM may be the same QTL and hence *qChlb 5D* is validated in this mapping population. The other two QTL detected for this trait were not reported earlier at these locations and designated as *qChlb7.1* and *qChlb8.1* (Table 3; Fig. 9A).

The QTL, *qChla8.1* and *qChlb8.1* for Chl. a and Chl. b on chromosome 8 at 363 cM position were co-localized and located very closely. Another two QTL, *qPC8.2* and *qTSS8.2* on chromosome 8 at position 234 cM controlling protein and total soluble sugars content were found to be co-localized. Similarly, the QTL, *qSC11.1* and *qAmy11.1* starch and amylose content are significantly associated with marker, RM3701 and detected to be closely located at 48 cM position on the chromosome 11 (Table 3). This indicates that these pairs of characters will be inherited together to the progenies. In addition, these pair of traits showed strong positive correlation and hence easy for improvement in the breeding programs. Similar findings were reported in earlier mapping studies for high temperature tolerance, protein, iron, zinc content, iron toxicity tolerance, seedling vigour and antioxidant content in rice [2, 38, 45, 68–70].

## Conclusion

A wide genetic variation for protein, starch, amylose and total soluble sugars and chlorophyll content were observed in the germplasm lines used for association study. The prospectus donor lines carrying higher content of these biochemical traits were identified. The STRUCTURE software classified the representative population into 3 genetic structure groups. Specific allele frequency, gene diversity, informative markers and other diversity parameters estimated from the population in the panel population using 136 SSR markers. Various groups and sub-groups obtained from the population showed relationship within the members for their biochemical traits. Linkage disequilibrium was detected in the studied population for the six biochemical traits. Previously reported QTL, *qProt1*, *qPC6.2*, and *qPC8.2* for protein content; *qTSS8.1* for total soluble sugar; *qAC1.2* for amylose content; *qCH2* and *qSLCHH* for chlorophyll a while *qChl5D* for chlorophyll b were validated in this study. A total of 13 QTL controlling total protein content *qPC1.2*; *qTSS7.1*, *qTSS8.2* and *qTSS12.1* for total soluble sugars; *qSC2.1*, *qSC2.2*, *qSC6.1* and *qSC11.1* for starch content; *qAC11.1*, *qAC11.2* and *qAC11.3* for amylose content; *qChla8.1* for chlorophyll a content and *qChlb7.1* and *qChlb8.1* chlorophyll b detected by both Generalized Linear Model and Mixed Linear Model were detected as novel QTL. Co-localization of QTL, *qChla8.1* with *qChlb8.1* for Chl. a and Chl. b; *qPC8.2* and *qTSS8.2* for protein content and total soluble sugars while *qSC11.1* and *qAmy11.1* for starch and amylose content were observed in the study. The validated, co-localized and the novel QTL detected in this study will be useful for improvement of protein, starch, amylose, total soluble sugars and chlorophyll content in rice.

## Supplementary Information

**Additional file 1: Supplementary Fig. 1.**

**Additional file 2: Supplementary Fig. 2.**

**Additional file 3: Supplementary Table 1**. Mean estimates of chlorophyll a, chlorophyll b, starch, amylose, total protein andtotal soluble sugars content in the initial shortlisted population containing of 274 germplasm lines.

**Additional file 4: Supplementary Table 2**. Markers information of the selected 136 SSR markers used in the genotyping of 120 rice landraces.

**Additional file 5: Supplementary Table 3.** Assessment of genetic diversity parameters of the panel population containing 120 rice landraces using 136 SSR markers loci.

**Additional file 6: Supplementary Table 4.** Genetic structure ancestry value at K = 3 and classification of the panel population containing 120 landraces based on the biochemical traits.

**Additional file 7: Supplementary Table 5**. Significant marker-trait associations detected for chlorophyll a, chlorophyll b, starch, amylose, total protein and total soluble sugars by GLM approach at *p* < 0.01.

**Additional file 8: Supplementary Table 6**. Significant marker-trait associations detected for chlorophyll a, chlorophyll b, starch, amylose, total protein and total soluble sugars by MLM approach at *p* < 0.01.

## Data Availability

The data generated or analyzed in this study are included in this article.
