# Supplementary figures and images for: Association mapping for protein, total soluble sugars, starch, amylose and chlorophyll content in rice
Source: BMC Plant Biol. 2022 Dec 29;22:620. doi: 10.1186/s12870-022-04015-8 (PMC9801606; doi:10.1186/s12870-022-04015-8)

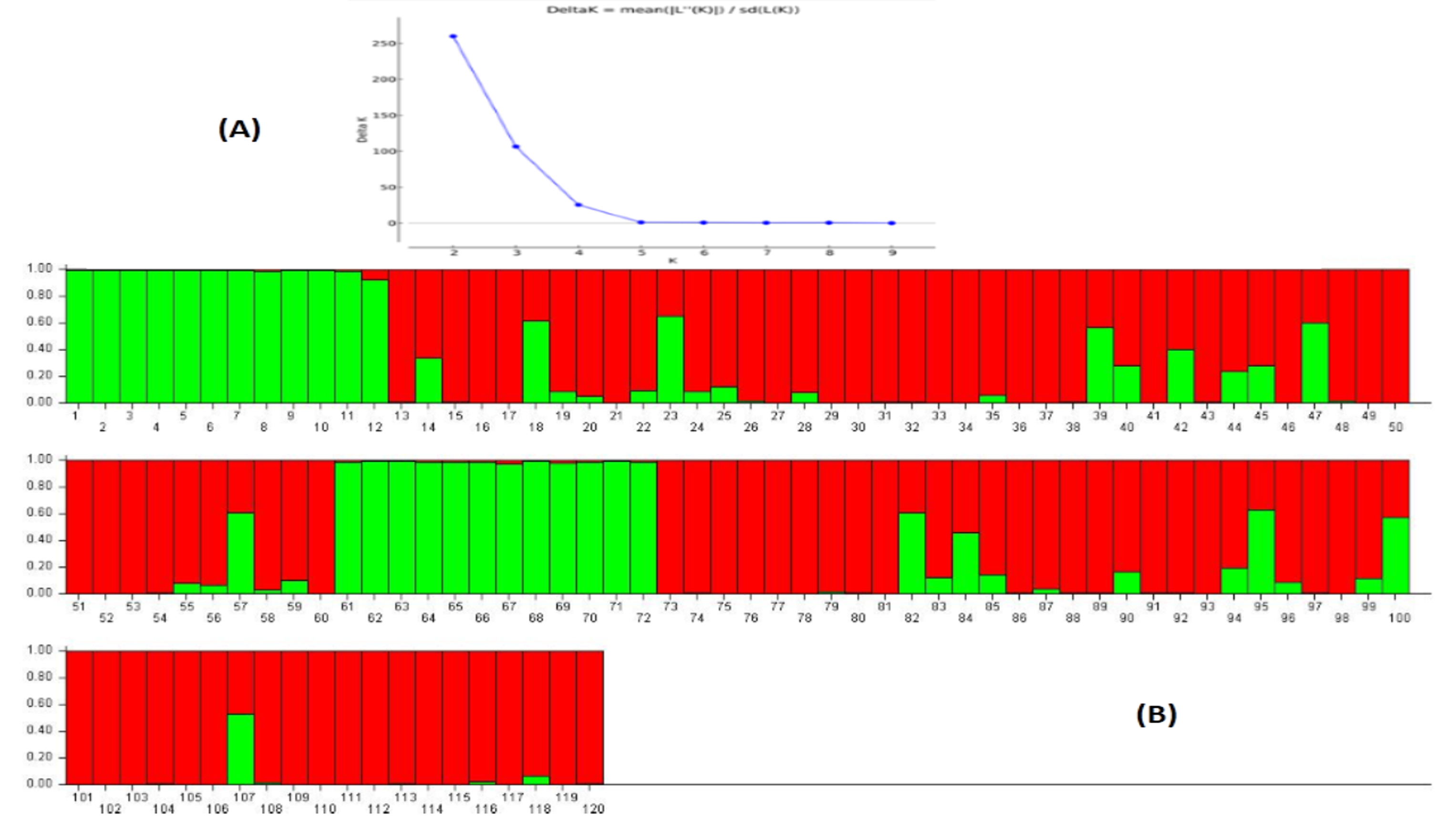

Supplement: Supplementary file 1 — Additional file 1: Supplementary Fig. 1. [file 12870_2022_4015_MOESM1_ESM.tif]

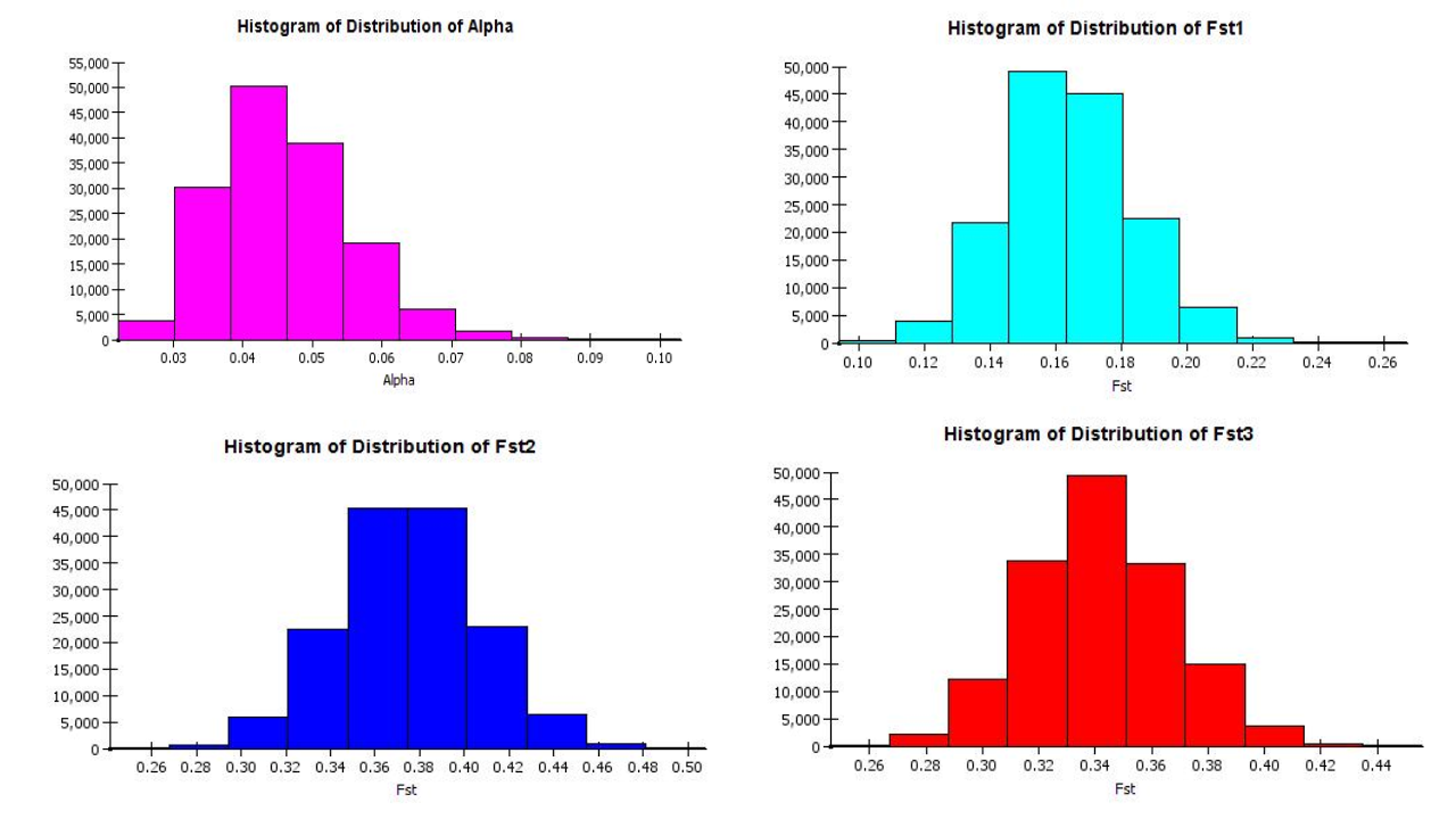

Supplement: Supplementary file 2 — Additional file 2: Supplementary Fig. 2. [file 12870_2022_4015_MOESM2_ESM.tif]
