# Supplementary material for: Association mapping for protein, total soluble sugars, starch, amylose and chlorophyll content in rice
Source: BMC Plant Biol. 2022 Dec 29;22:620. doi: 10.1186/s12870-022-04015-8 (PMC9801606; doi:10.1186/s12870-022-04015-8)
Supplement: Supplementary file 3 — Additional file 3: Supplementary Table 1. Mean estimates of chlorophyll a, chlorophyll b, starch, amylose, total protein andtotal soluble sugars content in the initial shortlisted population containing of 274 germplasm lines. [file 12870_2022_4015_MOESM3_ESM.docx]

**Supplementary Table 1**. Mean estimates of chlorophyll a, chlorophyll b, starch, amylose, total protein andtotal soluble sugars

content in the initial shortlisted population containingof 274germplasm lines.

| **SL NO** | **Name of the landrace/germplasm** | **Chl a (mg/g)** | **Chl b (mg/g)** | **Starch (%)** | **Amylose (%)** | **TP (%)** | **TSS (%)** |
| --- | --- | --- | --- | --- | --- | --- | --- |
| 1 | PhourelAngoubi | 1.690 | 0.080 | 50.210 | 19.230 | 5.050 | 0.00354 |
| 2 | Changlei | 1.750 | 0.120 | 53.450 | 20.250 | 4.030 | 0.00365 |
| 3 | Napchong | 0.060 | 0.190 | 55.630 | 18.630 | 0.230 | 0.00421 |
| 4 | Badadhan | 0.130 | 0.280 | 68.250 | 21.250 | 5.460 | 0.00372 |
| 5 | WangaBarugulu | 0.160 | 1.250 | 49.580 | 22.160 | 6.260 | 0.00381 |
| 6 | Ramakoli | 0.180 | 1.360 | 58.740 | 25.360 | 5.230 | 0.00365 |
| 7 | Maguramanji | 0.450 | 0.590 | 55.260 | 19.260 | 4.120 | 0.00497 |
| 8 | Airwerega | 1.230 | 0.360 | 67.250 | 22.250 | 7.360 | 0.00512 |
| 9 | ManoharSali | 0.399 | 0.225 | 69.145 | 25.605 | 8.065 | 0.00487 |
| 10 | Bengalijoha | 0.368 | 0.226 | 63.920 | 25.800 | 11.285 | 0.00456 |
| 11 | Anapachidhan | 0.761 | 0.451 | 64.725 | 25.260 | 7.575 | 0.00647 |
| 12 | Umamata | 0.860 | 0.250 | 52.230 | 20.250 | 8.250 | 0.00362 |
| 13 | Rohidhan-1 | 1.350 | 0.340 | 43.650 | 19.360 | 8.120 | 0.00524 |
| 14 | Gerwathor | 1.230 | 0.220 | 52.120 | 23.250 | 7.690 | 0.00623 |
| 15 | Jira | 5.685 | 3.543 | 80.215 | 25.585 | 3.305 | 0.00349 |
| 16 | Sonamasuri | 2.656 | 1.533 | 72.835 | 22.775 | 4.880 | 0.00475 |
| 17 | Kanakchampa | 0.127 | 0.113 | 72.500 | 24.620 | 10.380 | 0.00485 |
| 18 | Kaikee | 1.140 | 0.090 | 44.230 | 20.140 | 8.420 | 0.00452 |
| 19 | Champalidhan | 0.336 | 0.226 | 63.570 | 23.330 | 10.915 | 0.00486 |
| 20 | Ahimachutki | 0.463 | 0.293 | 89.975 | 21.610 | 10.940 | 0.01064 |
| 21 | Ampang | 0.507 | 0.316 | 54.575 | 25.055 | 11.450 | 0.00711 |
| 22 | Manaharrathori | 2.450 | 0.250 | 85.620 | 23.250 | 7.610 | 0.00412 |
| 23 | Ganorhibuna | 1.560 | 0.360 | 43.540 | 19.250 | 6.230 | 0.00316 |
| 24 | Kadamful | 0.890 | 0.250 | 40.520 | 16.350 | 5.980 | 0.00462 |
| 25 | Bakuldhan | 0.780 | 0.980 | 54.360 | 18.240 | 7.360 | 0.00412 |
| 26 | Kusumal | 1.916 | 0.452 | 63.270 | 23.130 | 8.915 | 0.00765 |
| 27 | Phongangangamphou | 1.033 | 0.723 | 26.045 | 23.265 | 11.280 | 0.00432 |
| 28 | Langmanbu | 1.971 | 1.379 | 63.195 | 23.475 | 13.835 | 0.00493 |
| 29 | Palina dhan-2 | 0.650 | 0.680 | 51.410 | 19.250 | 7.590 | 0.00523 |
| 30 | AC-6006 | 0.970 | 0.130 | 54.360 | 18.450 | 8.430 | 0.00512 |
| 31 | AC-6617 | 1.090 | 1.120 | 56.230 | 19.250 | 7.630 | 0.00496 |
| 32 | AC-7009 | 1.350 | 1.360 | 55.290 | 20.230 | 8.120 | 0.00487 |
| 33 | AC-7124 | 2.680 | 0.690 | 52.150 | 21.140 | 7.250 | 0.00423 |
| 34 | AC-7204 | 0.690 | 0.890 | 64.230 | 22.350 | 4.350 | 0.00369 |
| 35 | AC-10333 | 2.370 | 0.430 | 60.360 | 19.560 | 6.890 | 0.00341 |
| 36 | AC-10438 | 1.270 | 0.110 | 67.230 | 18.250 | 7.110 | 0.00335 |
| 37 | Kalimekri77-5 | 0.418 | 0.214 | 87.285 | 20.730 | 7.685 | 0.00604 |
| 38 | PMK2 | 0.380 | 0.306 | 70.330 | 22.490 | 5.950 | 0.00499 |
| 39 | Jyothi | 0.690 | 0.160 | 68.450 | 20.260 | 7.280 | 0.00412 |
| 40 | Charnumbery | 0.780 | 0.230 | 61.250 | 21.350 | 6.230 | 0.00425 |
| 41 | Shahiram | 0.840 | 0.270 | 65.230 | 20.450 | 6.140 | 0.00392 |
| 42 | Sonal | 0.870 | 0.690 | 74.250 | 26.230 | 5.420 | 0.00412 |
| 43 | Laxmibilash | 1.011 | 1.651 | 67.780 | 24.220 | 10.005 | 0.00401 |
| 44 | Lalgundi | 0.190 | 0.216 | 62.985 | 25.950 | 14.060 | 0.00448 |
| 45 | Magra | 0.139 | 0.193 | 66.175 | 24.045 | 10.640 | 0.00411 |
| 46 | Padma rekha | 0.890 | 0.890 | 59.300 | 25.120 | 5.490 | 0.00468 |
| 47 | Latamahu | 0.438 | 0.145 | 68.210 | 22.985 | 7.585 | 0.00368 |
| 48 | Kundadhan | 0.558 | 0.384 | 94.020 | 20.900 | 11.460 | 0.00633 |
| 49 | Karpurkanti | 0.908 | 0.152 | 67.215 | 24.040 | 6.815 | 0.00315 |
| 50 | Gandhasali | 1.230 | 0.790 | 50.180 | 24.320 | 6.130 | 0.00432 |
| 51 | Pintumasuri | 1.190 | 0.640 | 46.450 | 19.250 | 6.030 | 0.00675 |
| 52 | Alkachuri | 0.780 | 1.110 | 56.230 | 23.460 | 7.210 | 0.00841 |
| 53 | Mahamaga | 0.501 | 0.373 | 87.905 | 24.125 | 14.565 | 0.00381 |
| 54 | Jhingesal | 1.078 | 0.654 | 89.605 | 25.395 | 7.470 | 0.00477 |
| 55 | Gochi | 0.272 | 0.330 | 84.040 | 23.240 | 12.080 | 0.00622 |
| 56 | Chatuimuchi | 1.382 | 0.835 | 64.130 | 25.140 | 10.600 | 0.00432 |
| 57 | Amonabao | 0.890 | 1.680 | 53.210 | 22.150 | 6.580 | 0.00312 |
| 58 | Annapurna | 0.560 | 1.490 | 51.260 | 21.150 | 7.610 | 0.00651 |
| 59 | Assambiroin | 1.230 | 2.000 | 55.640 | 20.540 | 5.260 | 0.00798 |
| 60 | Balam | 1.160 | 56.000 | 52.120 | 25.120 | 7.570 | 0.00821 |
| 61 | Bambi mugai | 1.140 | 2.370 | 64.180 | 23.160 | 7.360 | 0.00811 |
| 62 | Barbali | 1.650 | 1.000 | 66.420 | 22.450 | 7.120 | 0.00862 |
| 63 | Barhasal | 1.690 | 29.000 | 64.350 | 21.410 | 8.690 | 0.00912 |
| 64 | Baskati | 2.430 | 0.650 | 65.360 | 23.460 | 8.120 | 0.01020 |
| 65 | Baula | 0.960 | 0.470 | 69.670 | 22.180 | 8.450 | 0.00842 |
| 66 | Gondiachampeisiali | 0.787 | 0.187 | 85.015 | 22.525 | 11.200 | 0.00433 |
| 67 | Gandhakasala | 0.076 | 0.228 | 66.130 | 24.715 | 10.030 | 0.00575 |
| 68 | D1 | 0.279 | 0.215 | 84.695 | 22.985 | 13.940 | 0.00453 |
| 69 | Bhasakalma | 0.940 | 0.490 | 70.160 | 20.190 | 8.690 | 0.00652 |
| 70 | Kantakaamala | 1.142 | 0.161 | 94.820 | 19.610 | 9.125 | 0.00568 |
| 71 | Jyothi | 0.716 | 0.474 | 86.405 | 23.060 | 13.905 | 0.00469 |
| 72 | Marathondi | 0.849 | 0.599 | 84.085 | 22.760 | 15.090 | 0.00567 |
| 73 | Chhotadahiya | 1.180 | 0.480 | 58.240 | 23.120 | 7.590 | 0.00432 |
| 74 | Chingair | 2.310 | 0.270 | 59.890 | 21.140 | 7.630 | 0.00582 |
| 75 | Dhanigoda | 1.460 | 0.650 | 62.650 | 20.180 | 9.250 | 0.00562 |
| 76 | Moirangphon | 1.083 | 0.803 | 62.275 | 25.145 | 9.450 | 0.00484 |
| 77 | Chakhaosimpak | 0.893 | 0.679 | 64.885 | 25.575 | 9.790 | 0.00517 |
| 78 | Kartiksal | 2.301 | 1.341 | 94.195 | 22.655 | 14.140 | 0.00277 |
| 79 | Gandhi biroin | 1.140 | 0.430 | 50.450 | 21.190 | 9.150 | 0.00512 |
| 80 | Gengene | 0.780 | 0.120 | 53.650 | 21.180 | 8.120 | 0.00498 |
| 81 | Hermanona | 0.890 | 0.150 | 51.890 | 20.150 | 8.760 | 0.00365 |
| 82 | Hugla | 0.760 | 0.260 | 61.250 | 21.140 | 7.620 | 0.00412 |
| 83 | Jool | 0.740 | 0.350 | 52.890 | 22.150 | 7.280 | 0.00435 |
| 84 | Kakharua | 1.230 | 0.890 | 55.570 | 21.190 | 6.520 | 0.00465 |
| 85 | Kaksal | 0.980 | 0.760 | 46.870 | 22.230 | 6.450 | 0.00419 |
| 86 | Kalakatki | 0.740 | 0.460 | 51.230 | 21.180 | 5.450 | 0.00478 |
| 87 | TKM10 | 0.291 | 0.341 | 82.795 | 26.790 | 4.955 | 0.00552 |
| 88 | Belimuruduga | 0.190 | 0.319 | 82.810 | 20.530 | 9.695 | 0.00398 |
| 89 | Kalokumara | 0.390 | 0.180 | 55.620 | 22.140 | 5.860 | 0.00489 |
| 90 | Karagagoda | 0.760 | 0.350 | 59.890 | 20.150 | 7.360 | 0.00518 |
| 91 | Karhani | 0.490 | 0.260 | 61.580 | 22.150 | 8.120 | 0.00523 |
| 92 | Vachaw | 0.608 | 0.395 | 71.225 | 24.565 | 10.630 | 0.00520 |
| 93 | Adira-3 | 0.932 | 0.610 | 86.645 | 24.125 | 12.705 | 0.00697 |
| 94 | Adira-1 | 0.557 | 0.533 | 94.215 | 21.980 | 14.210 | 0.00476 |
| 95 | Bharati | 0.120 | 0.251 | 91.080 | 23.705 | 18.255 | 0.00532 |
| 96 | Karnidhan | 0.750 | 0.240 | 62.350 | 21.140 | 8.960 | 0.00563 |
| 97 | Basumati-B | 0.139 | 0.159 | 68.180 | 24.405 | 9.605 | 0.00530 |
| 98 | Dadghani | 1.008 | 0.678 | 65.340 | 24.760 | 10.695 | 0.00468 |
| 99 | Baranga | 1.478 | 0.399 | 90.850 | 20.730 | 12.425 | 0.00550 |
| 100 | Katraibhog | 0.320 | 0.190 | 63.890 | 20.180 | 7.950 | 0.00612 |
| 101 | Kelash 1981 | 1.120 | 1.110 | 45.230 | 21.150 | 5.230 | 0.00625 |
| 102 | Koya 4 | 1.050 | 1.250 | 45.120 | 22.130 | 5.460 | 0.00632 |
| 103 | Dudhamani | 0.728 | 0.669 | 71.195 | 27.010 | 9.930 | 0.00493 |
| 104 | Mahipaljeera | 2.094 | 0.588 | 84.090 | 21.160 | 11.065 | 0.00687 |
| 105 | Batachudi | 1.948 | 0.555 | 64.795 | 24.495 | 7.405 | 0.00994 |
| 106 | Salati | 1.358 | 0.342 | 89.705 | 20.410 | 11.865 | 0.00611 |
| 107 | Koya ho baba | 1.160 | 0.890 | 26.560 | 23.450 | 6.250 | 0.00587 |
| 108 | Lalbora | 0.680 | 0.940 | 89.320 | 23.470 | 6.350 | 0.00998 |
| 109 | Shayam | 1.160 | 0.745 | 61.540 | 23.705 | 11.585 | 0.00347 |
| 110 | Jhagrikartik | 0.247 | 0.215 | 61.485 | 24.215 | 10.590 | 0.00441 |
| 111 | Liktimachi | 1.586 | 0.489 | 43.930 | 24.760 | 10.430 | 0.00486 |
| 112 | Langalmuthi | 0.980 | 0.930 | 72.620 | 23.580 | 6.120 | 0.00528 |
| 113 | Likekakua | 0.760 | 0.920 | 64.350 | 22.160 | 7.150 | 0.00536 |
| 114 | Marchal | 1.140 | 1.120 | 57.360 | 22.280 | 7.110 | 0.00653 |
| 115 | Meghi | 0.960 | 1.080 | 56.350 | 23.240 | 6.980 | 0.00425 |
| 116 | Mornodoiga | 0.850 | 1.060 | 54.260 | 23.560 | 6.890 | 0.00468 |
| 117 | Motarmala | 0.810 | 0.690 | 56.690 | 24.180 | 7.230 | 0.00458 |
| 118 | Mugai | 0.820 | 0.890 | 58.560 | 21.130 | 7.210 | 0.00357 |
| 119 | Mugai | 0.750 | 0.120 | 46.650 | 21.010 | 8.210 | 0.00436 |
| 120 | Nagheri | 0.650 | 0.230 | 67.850 | 21.160 | 6.250 | 0.00521 |
| 121 | Nagheri | 1.230 | 0.360 | 56.450 | 19.540 | 6.440 | 0.00532 |
| 122 | Radhabati | 1.605 | 0.523 | 69.795 | 24.270 | 9.835 | 0.00671 |
| 123 | Tikichudi | 2.296 | 0.860 | 87.475 | 21.515 | 12.375 | 0.00542 |
| 124 | Lalmunduria | 1.770 | 0.568 | 81.465 | 21.740 | 12.460 | 0.00493 |
| 125 | Nalbora | 1.210 | 0.250 | 71.450 | 21.160 | 7.150 | 0.00589 |
| 126 | Neta | 1.130 | 1.080 | 70.650 | 20.150 | 7.120 | 0.00632 |
| 127 | Netakalani | 0.780 | 0.980 | 52.340 | 22.320 | 5.260 | 0.00289 |
| 128 | Nirjhara | 2.160 | 1.250 | 56.450 | 22.120 | 5.360 | 0.00365 |
| 129 | Palbari | 2.110 | 2.120 | 67.480 | 20.150 | 7.620 | 0.00254 |
| 130 | Kalame | 1.864 | 1.139 | 72.400 | 20.610 | 9.020 | 0.00361 |
| 131 | Lusai | 3.784 | 2.324 | 70.795 | 23.825 | 3.600 | 0.00449 |
| 132 | Malbar | 3.030 | 1.804 | 91.760 | 21.350 | 9.150 | 0.00724 |
| 133 | Bilijaya | 2.060 | 1.149 | 68.345 | 26.525 | 7.135 | 0.00440 |
| 134 | Magura-s | 0.177 | 0.147 | 54.730 | 23.785 | 12.275 | 0.00396 |
| 135 | Panati | 1.340 | 1.070 | 66.790 | 21.150 | 5.360 | 0.00698 |
| 136 | PB140 | 0.590 | 1.650 | 81.250 | 20.140 | 8.120 | 0.00425 |
| 137 | Rajesh | 0.420 | 1.430 | 86.350 | 23.240 | 7.980 | 0.00575 |
| 138 | Cheruvirippu | 0.463 | 0.305 | 86.655 | 22.765 | 12.805 | 0.00416 |
| 139 | Sugandha-2 | 0.773 | 0.588 | 71.160 | 23.775 | 9.435 | 0.00487 |
| 140 | Uttarbangalocal-9 | 0.234 | 0.261 | 59.185 | 26.045 | 9.595 | 0.00327 |
| 141 | Palinadhan-1 | 0.298 | 0.306 | 71.530 | 25.450 | 7.865 | 0.00461 |
| 142 | BodiKaberi | 1.409 | 0.365 | 83.255 | 20.770 | 9.855 | 0.00754 |
| 143 | Barda | 1.510 | 0.456 | 67.900 | 25.020 | 7.730 | 0.00619 |
| 144 | RPHP112 | 0.320 | 1.090 | 64.790 | 21.150 | 7.650 | 0.00571 |
| 145 | Saathi | 0.680 | 0.580 | 66.230 | 20.150 | 8.360 | 0.00597 |
| 146 | Setka 36 | 0.490 | 0.510 | 62.450 | 21.150 | 8.120 | 0.00546 |
| 147 | Sugandha | 0.510 | 0.230 | 59.450 | 21.160 | 7.650 | 0.00282 |
| 148 | Urebanga | 0.470 | 0.460 | 61.120 | 21.180 | 7.340 | 0.00589 |
| 149 | Vutmari | 0.640 | 0.230 | 46.530 | 22.450 | 9.560 | 0.00546 |
| 150 | Geda | 0.630 | 0.210 | 52.590 | 21.450 | 9.120 | 0.00523 |
| 151 | Chudi | 2.036 | 0.829 | 81.685 | 22.080 | 13.405 | 0.00516 |
| 152 | Jhitikuji | 2.150 | 0.736 | 60.300 | 25.385 | 8.850 | 0.00598 |
| 153 | Pondremunduria | 2.119 | 0.690 | 88.985 | 22.030 | 12.975 | 0.00541 |
| 154 | Phoudum | 0.691 | 0.532 | 65.975 | 23.515 | 9.290 | 0.00576 |
| 155 | Taothali | 0.964 | 0.529 | 63.525 | 27.390 | 9.305 | 0.00599 |
| 156 | Haldigundi | 0.640 | 0.450 | 63.210 | 20.450 | 9.110 | 0.00602 |
| 157 | Saragadebangi | 1.180 | 0.460 | 71.120 | 22.150 | 8.540 | 0.00615 |
| 158 | Kadara | 1.120 | 0.460 | 51.230 | 21.160 | 8.130 | 0.00616 |
| 159 | Sundarmadei | 2.140 | 0.780 | 45.360 | 23.240 | 8.140 | 0.00625 |
| 160 | Mayangkhang-I | 2.624 | 1.808 | 93.220 | 19.970 | 12.710 | 0.00473 |
| 161 | Aujari | 3.638 | 2.302 | 59.760 | 23.570 | 10.055 | 0.00436 |
| 162 | Chingforechokua | 0.285 | 0.181 | 62.785 | 29.560 | 10.225 | 0.00407 |
| 163 | Tilibora | 0.285 | 0.203 | 61.210 | 23.560 | 10.185 | 0.00339 |
| 164 | Kanaimuluk | 0.139 | 0.136 | 68.495 | 25.800 | 8.310 | 0.00484 |
| 165 | Mikirahu | 0.190 | 0.113 | 58.395 | 22.735 | 10.315 | 0.01121 |
| 166 | Pratao | 0.342 | 0.295 | 97.805 | 21.480 | 11.255 | 0.00582 |
| 167 | Sundargada | 1.560 | 0.410 | 57.230 | 21.150 | 6.580 | 0.00619 |
| 168 | Kala dhusura | 1.780 | 1.080 | 46.560 | 24.150 | 6.590 | 0.00622 |
| 169 | Godhikhejara | 0.980 | 0.980 | 77.150 | 25.260 | 7.120 | 0.00725 |
| 170 | Laxmikajal | 0.680 | 1.650 | 75.140 | 21.230 | 7.130 | 0.00715 |
| 171 | Mugei | 0.490 | 1.050 | 72.230 | 20.150 | 7.480 | 0.00763 |
| 172 | Mugi | 0.280 | 0.590 | 78.680 | 19.560 | 7.190 | 0.00653 |
| 173 | Jhumpurimalata | 1.340 | 0.480 | 68.590 | 23.210 | 7.120 | 0.00558 |
| 174 | Sal jhantri | 0.690 | 0.440 | 69.230 | 21.130 | 7.140 | 0.00682 |
| 175 | Aditya | 0.335 | 0.432 | 83.920 | 19.550 | 8.525 | 0.00505 |
| 176 | Noorthipathu | 0.596 | 0.441 | 92.305 | 21.230 | 13.510 | 0.00761 |
| 177 | Tulasi | 2.789 | 1.726 | 90.385 | 22.410 | 13.110 | 0.00475 |
| 178 | MDU-5 | 0.425 | 0.294 | 66.455 | 22.535 | 8.335 | 0.00396 |
| 179 | Nathmohan | 0.560 | 0.410 | 70.150 | 21.150 | 7.130 | 0.00587 |
| 180 | Hundamakara | 0.740 | 0.420 | 65.230 | 20.150 | 6.890 | 0.00526 |
| 181 | Memabalbok | 0.748 | 0.371 | 64.050 | 25.490 | 11.705 | 0.00386 |
| 182 | Mimagisim | 0.374 | 0.237 | 55.750 | 25.125 | 10.265 | 0.00694 |
| 183 | Mimahambel | 1.014 | 0.678 | 67.980 | 19.615 | 11.400 | 0.00655 |
| 184 | Latachaunri | 0.520 | 0.167 | 93.285 | 20.470 | 10.595 | 0.00598 |
| 185 | Champaeisiali | 0.438 | 0.145 | 91.775 | 21.265 | 10.305 | 0.00618 |
| 186 | Kathidhan | 0.056 | 0.332 | 86.165 | 23.340 | 9.770 | 0.00488 |
| 187 | Dhobakakiri | 0.230 | 0.260 | 64.250 | 22.130 | 6.540 | 0.00456 |
| 188 | Boula | 0.480 | 0.350 | 68.320 | 24.150 | 7.360 | 0.00458 |
| 189 | Kaniar | 0.901 | 0.186 | 85.095 | 21.935 | 12.670 | 0.00566 |
| 190 | Balisaralaktimachi-k | 0.216 | 0.124 | 58.150 | 23.380 | 9.490 | 0.00404 |
| 191 | Landi | 1.517 | 0.318 | 89.285 | 22.400 | 10.790 | 0.00571 |
| 192 | Sundarbhojna | 0.190 | 0.220 | 66.350 | 26.350 | 5.680 | 0.00424 |
| 193 | Kuliha | 0.230 | 0.210 | 48.690 | 25.410 | 4.320 | 0.00421 |
| 194 | Hajirimala | 2.650 | 0.130 | 47.250 | 22.320 | 4.560 | 0.00425 |
| 195 | Nadiarasa | 0.690 | 0.450 | 51.240 | 23.410 | 7.590 | 0.00625 |
| 196 | Kalanpati | 0.780 | 0.240 | 55.230 | 23.450 | 8.120 | 0.00623 |
| 197 | Majhi | 0.890 | 0.170 | 59.620 | 23.460 | 4.630 | 0.00589 |
| 198 | Manavari | 0.501 | 0.373 | 99.495 | 21.115 | 8.035 | 0.00585 |
| 199 | Pandya | 2.656 | 1.578 | 98.795 | 22.305 | 7.595 | 0.00750 |
| 200 | Badra | 2.561 | 1.511 | 95.650 | 20.490 | 10.580 | 0.00456 |
| 201 | Kalajeri | 0.520 | 0.130 | 57.230 | 22.150 | 7.120 | 0.00621 |
| 202 | Majhi | 0.460 | 0.180 | 56.280 | 22.450 | 6.810 | 0.00652 |
| 203 | Sunakathi | 0.370 | 0.190 | 52.360 | 21.150 | 6.490 | 0.00754 |
| 204 | Kalinga-2 | 0.290 | 0.220 | 59.340 | 21.160 | 6.580 | 0.00787 |
| 205 | Kalinga-3 | 0.140 | 1.050 | 63.230 | 22.350 | 7.350 | 0.00745 |
| 206 | Sahabhagidhan | 0.360 | 1.060 | 61.280 | 19.260 | 7.190 | 0.00629 |
| 207 | Hazaridhan | 1.390 | 1.110 | 63.250 | 20.150 | 7.290 | 0.00603 |
| 208 | Satabdi | 1.130 | 0.980 | 66.250 | 21.750 | 7.680 | 0.00714 |
| 209 | Swarna | 0.520 | 0.950 | 67.590 | 22.130 | 8.120 | 0.00512 |
| 210 | Naveen | 0.790 | 0.850 | 77.280 | 20.160 | 8.110 | 0.00716 |
| 211 | Satyabhama | 0.360 | 0.260 | 68.950 | 24.130 | 7.840 | 0.00718 |
| 212 | Kaberi | 1.396 | 0.376 | 64.255 | 25.235 | 9.310 | 0.00598 |
| 213 | Kakudimanji | 1.402 | 0.342 | 59.995 | 25.790 | 6.680 | 0.00507 |
| 214 | Phourrel | 4.931 | 3.241 | 60.095 | 24.720 | 11.780 | 0.00468 |
| 215 | Gitanjali | 0.250 | 0.240 | 46.560 | 21.250 | 6.850 | 0.00258 |
| 216 | Crdhan-305 | 0.360 | 0.250 | 52.350 | 23.210 | 6.250 | 0.00842 |
| 217 | Crdhan-310 | 0.470 | 0.230 | 52.310 | 21.150 | 7.680 | 0.00958 |
| 218 | Crdhan-907 | 0.690 | 0.320 | 69.650 | 20.150 | 7.980 | 0.00789 |
| 219 | Govindbhog | 1.190 | 0.340 | 62.650 | 20.160 | 8.140 | 0.00546 |
| 220 | Nuakalazeera | 0.460 | 0.360 | 66.850 | 23.120 | 8.110 | 0.00587 |
| 221 | Chinamal | 0.641 | 0.074 | 68.775 | 25.415 | 8.785 | 0.00440 |
| 222 | Sreyas | 0.330 | 0.226 | 84.450 | 23.550 | 14.080 | 0.00373 |
| 223 | Pk-21 | 0.482 | 0.316 | 86.570 | 21.880 | 15.955 | 0.00615 |
| 224 | Jaya | 0.165 | 0.113 | 58.200 | 35.040 | 12.430 | 0.00378 |
| 225 | Masuri | 0.630 | 0.280 | 62.450 | 22.160 | 8.120 | 0.00623 |
| 226 | Heera | 1.140 | 0.640 | 53.150 | 22.180 | 7.630 | 0.00689 |
| 227 | CRDhan-1014 | 1.120 | 0.620 | 58.190 | 21.150 | 7.250 | 0.00368 |
| 228 | Nuadhusua | 0.280 | 0.820 | 47.280 | 22.160 | 9.480 | 0.00358 |
| 229 | Banskathi | 0.250 | 0.770 | 64.780 | 21.180 | 9.650 | 0.00687 |
| 230 | Kakchengphou | 0.481 | 0.431 | 63.150 | 24.555 | 13.110 | 0.00452 |
| 231 | Chingphou | 3.073 | 2.330 | 19.200 | 21.120 | 11.810 | 0.00467 |
| 232 | Phoaujaarangbele | 2.813 | 2.058 | 66.975 | 24.480 | 9.115 | 0.00473 |
| 233 | Turnaianganba | 1.229 | 0.950 | 65.975 | 25.330 | 8.785 | 0.00634 |
| 234 | Hanseswari | 0.230 | 0.620 | 63.650 | 22.160 | 6.580 | 0.00397 |
| 235 | Savitri | 1.170 | 0.610 | 62.150 | 22.490 | 7.110 | 0.00389 |
| 236 | Dhalaheera | 0.360 | 0.560 | 51.360 | 21.150 | 8.680 | 0.00417 |
| 237 | Mahulata | 0.250 | 0.520 | 59.480 | 19.250 | 7.590 | 0.00425 |
| 238 | Padmini | 0.940 | 0.480 | 57.580 | 18.460 | 8.450 | 0.00698 |
| 239 | Changli | 2.192 | 1.629 | 64.155 | 25.080 | 11.495 | 0.00503 |
| 240 | Mayangkhang-II | 1.160 | 0.779 | 59.300 | 24.430 | 11.400 | 0.00432 |
| 241 | Ratna | 0.980 | 0.460 | 56.150 | 23.160 | 7.980 | 0.00487 |
| 242 | Annada | 0.950 | 0.410 | 62.160 | 22.180 | 5.630 | 0.00354 |
| 243 | MayurKantha | 1.210 | 0.230 | 63.150 | 22.650 | 4.350 | 0.00362 |
| 244 | Gomati Dhan | 1.360 | 0.350 | 64.650 | 19.560 | 5.360 | 0.00412 |
| 245 | Kozhivalan | 0.811 | 0.451 | 84.325 | 23.045 | 14.505 | 0.00574 |
| 246 | PK6 | 0.393 | 0.271 | 83.715 | 23.505 | 15.160 | 0.00424 |
| 247 | Adira-2 | 1.040 | 0.632 | 83.130 | 21.515 | 14.210 | 0.00616 |
| 248 | Joha | 0.177 | 0.170 | 64.090 | 22.275 | 10.650 | 0.00383 |
| 249 | Lalat | 1.110 | 0.320 | 63.150 | 23.240 | 6.570 | 0.00528 |
| 250 | Chinikamini | 1.010 | 0.410 | 61.150 | 22.180 | 7.120 | 0.00587 |
| 251 | CR Dhan -801 | 1.320 | 0.510 | 62.650 | 22.560 | 7.190 | 0.00458 |
| 252 | Koompallai | 1.642 | 0.821 | 66.310 | 22.530 | 8.050 | 0.00564 |
| 253 | karinellu | 0.246 | 0.524 | 89.745 | 20.375 | 10.870 | 0.00628 |
| 254 | Pratikshya | 0.230 | 0.260 | 82.140 | 20.560 | 8.450 | 0.00429 |
| 255 | Lalat Mas | 0.740 | 0.280 | 66.520 | 22.210 | 7.590 | 0.00378 |
| 256 | Luna | 0.460 | 0.340 | 74.620 | 21.180 | 6.480 | 0.00398 |
| 257 | Abhirman | 0.850 | 1.240 | 78.850 | 23.450 | 6.580 | 0.00325 |
| 258 | kapanthi | 0.723 | 0.131 | 39.600 | 45.175 | 5.560 | 0.00594 |
| 259 | Kantakapura | 1.035 | 0.151 | 95.280 | 21.235 | 10.585 | 0.00630 |
| 260 | Ezhoml-2 | 0.545 | 0.396 | 87.805 | 22.255 | 13.995 | 0.00484 |
| 261 | Kalakanhu | 1.250 | 1.360 | 56.210 | 23.150 | 7.250 | 0.00365 |
| 262 | Pologada | 1.110 | 1.130 | 55.890 | 21.180 | 7.380 | 0.00625 |
| 263 | Majhi | 0.780 | 0.950 | 55.140 | 20.450 | 6.590 | 0.00415 |
| 264 | Rajapateni | 0.890 | 0.480 | 52.460 | 23.450 | 8.120 | 0.00458 |
| 265 | Moiranghouanganba | 2.409 | 1.180 | 63.940 | 24.600 | 9.580 | 0.00405 |
| 266 | Kabokphou | 1.293 | 0.893 | 60.305 | 23.705 | 11.300 | 0.00418 |
| 267 | Manipurlocal | 0.532 | 0.327 | 62.475 | 24.145 | 9.965 | 0.00437 |
| 268 | Dalipahata | 0.250 | 0.680 | 58.460 | 22.140 | 8.630 | 0.00365 |
| 269 | Kadara | 0.450 | 0.350 | 50.180 | 23.250 | 7.590 | 0.00752 |
| 270 | Nadiarasa | 0.360 | 0.310 | 56.540 | 22.180 | 5.260 | 0.00657 |
| 271 | Gouri | 3.385 | 1.995 | 68.155 | 25.410 | 4.430 | 0.00602 |
| 272 | Chitapa | 2.587 | 1.396 | 94.125 | 22.920 | 11.495 | 0.00782 |
| 273 | Bilipandya | 4.748 | 2.887 | 67.165 | 24.100 | 4.170 | 0.00632 |
| 274 | Jayapadma | 2.770 | 1.658 | 90.715 | 21.190 | 11.525 | 0.00390 |

Chl a: Chlorophyll a content; Chl b: Chlorophyll b content; Starch: Starch content; Amylose: Amylose content;

TP: Total protein content; TSS: Total soluble sugars content
