# Supplementary material for: Association mapping for protein, total soluble sugars, starch, amylose and chlorophyll content in rice
Source: BMC Plant Biol. 2022 Dec 29;22:620. doi: 10.1186/s12870-022-04015-8 (PMC9801606; doi:10.1186/s12870-022-04015-8)
Supplement: Supplementary file 5 — Additional file 5: Supplementary Table 3. Assessment of genetic diversity parameters of the panel population containing 120 rice landraces using 136 SSR markers loci. [file 12870_2022_4015_MOESM5_ESM.docx]

**Supplementary Table 3.** Assessment of genetic diversity parameters of the panel population containing 120 rice landraces using 136 SSR markers loci.

| **SL No** | **Marker** | **Number of Alleles** | **Major Allele**  **Frequency** | **Gene Diversity** | **Heterozygosity** | **PIC** | **Inbreeding Coefficient (f)** |
| --- | --- | --- | --- | --- | --- | --- | --- |
| 1 | RM5310 | 4 | 0.783 | 0.367 | 0.033 | 0.343 | 0.909 |
| 2 | RM582 | 4 | 0.708 | 0.465 | 0.033 | 0.432 | 0.928 |
| 3 | RM13335 | 4 | 0.562 | 0.531 | 0.008 | 0.434 | 0.984 |
| 4 | RM6275 | 4 | 0.720 | 0.446 | 0.058 | 0.411 | 0.870 |
| 5 | RM50 | 4 | 0.4 | 0.688 | 0.025 | 0.630 | 0.963 |
| 6 | RM85 | 4 | 0.412 | 0.675 | 0.125 | 0.614 | 0.816 |
| 7 | RM222 | 4 | 0.629 | 0.557 | 0.025 | 0.518 | 0.955 |
| 8 | RM247 | 5 | 0.5 | 0.596 | 0.066 | 0.518 | 0.889 |
| 9 | RM328 | 3 | 0.566 | 0.58 | 0 | 0.512 | 1 |
| 10 | RM337 | 6 | 0.445 | 0.668 | 0.116 | 0.612 | 0.826 |
| 11 | RM340 | 5 | 0.712 | 0.453 | 0.1 | 0.414 | 0.781 |
| 12 | RM470 | 5 | 0.462 | 0.690 | 0.833 | 0.644 | -0.202 |
| 13 | RM472 | 3 | 0.512 | 0.507 | 0.091 | 0.386 | 0.820 |
| 14 | RM506 | 3 | 0.683 | 0.459 | 0.133 | 0.390 | 0.711 |
| 15 | RM1812 | 3 | 0.441 | 0.606 | 0 | 0.522 | 1 |
| 16 | RM3701 | 4 | 0.675 | 0.484 | 0.491 | 0.428 | -0.011 |
| 17 | RM6947 | 3 | 0.883 | 0.211 | 0 | 0.199 | 1 |
| 18 | RM14978 | 3 | 0.416 | 0.639 | 0 | 0.562 | 1 |
| 19 | RM18776 | 3 | 0.845 | 0.267 | 0.025 | 0.242 | 0.907 |
| 20 | RM22034 | 3 | 0.916 | 0.155 | 0 | 0.147 | 1 |
| 21 | RM24161 | 4 | 0.541 | 0.612 | 0.116 | 0.552 | 0.810 |
| 22 | RM223 | 5 | 0.654 | 0.536 | 0.058 | 0.504 | 0.892 |
| 23 | RM440 | 5 | 0.408 | 0.689 | 0.258 | 0.634 | 0.627 |
| 24 | RM201 | 3 | 0.479 | 0.577 | 0.025 | 0.486 | 0.957 |
| 25 | RM216 | 4 | 0.512 | 0.639 | 0.125 | 0.583 | 0.805 |
| 26 | RM258 | 3 | 0.383 | 0.651 | 0 | 0.576 | 1 |
| 27 | RM286 | 4 | 0.470 | 0.632 | 0.1 | 0.562 | 0.843 |
| 28 | RM3735 | 4 | 0.333 | 0.725 | 0.958 | 0.673 | -0.317 |
| 29 | RM1347 | 3 | 0.516 | 0.566 | 0 | 0.474 | 1 |
| 30 | RM7571 | 3 | 0.712 | 0.433 | 0.008 | 0.373 | 0.980 |
| 31 | RM14723 | 4 | 0.491 | 0.643 | 0.2 | 0.581 | 0.691 |
| 32 | RM103 | 3 | 0.491 | 0.558 | 0.766 | 0.460 | -0.368 |
| 33 | RM315 | 3 | 0.866 | 0.235 | 0 | 0.214 | 1 |
| 34 | RM225 | 3 | 0.525 | 0.547 | 0.183 | 0.448 | 0.667 |
| 35 | RM486 | 3 | 0.654 | 0.468 | 0.108 | 0.379 | 0.770 |
| 36 | RM256 | 3 | 0.720 | 0.411 | 0.058 | 0.339 | 0.859 |
| 37 | RM1113 | 3 | 0.670 | 0.456 | 0.058 | 0.372 | 0.873 |
| 38 | RM3423 | 3 | 0.5 | 0.574 | 0 | 0.484 | 1 |
| 39 | RM6100 | 3 | 0.441 | 0.643 | 0.033 | 0.568 | 0.948 |
| 40 | RM590 | 3 | 0.725 | 0.430 | 0.066 | 0.384 | 0.846 |
| 41 | RM5793 | 3 | 0.633 | 0.524 | 0.016 | 0.463 | 0.968 |
| 42 | RM405 | 3 | 0.675 | 0.491 | 0 | 0.441 | 1 |
| 43 | RM547 | 5 | 0.470 | 0.573 | 0.166 | 0.481 | 0.711 |
| 44 | RM7364 | 5 | 0.620 | 0.573 | 0.166 | 0.540 | 0.711 |
| 45 | RM205 | 3 | 0.620 | 0.532 | 0.025 | 0.466 | 0.953 |
| 46 | RM167 | 4 | 0.704 | 0.462 | 0.1 | 0.421 | 0.785 |
| 47 | RM229 | 4 | 0.358 | 0.707 | 0.133 | 0.652 | 0.812 |
| 48 | RM20A | 3 | 0.625 | 0.533 | 0.016 | 0.471 | 0.968 |
| 49 | RM235 | 5 | 0.395 | 0.718 | 0.175 | 0.671 | 0.758 |
| 50 | RM7003 | 4 | 0.666 | 0.501 | 0.083 | 0.452 | 0.835 |
| 51 | RM5436 | 4 | 0.441 | 0.621 | 0.058 | 0.544 | 0.906 |
| 52 | RM25181 | 5 | 0.379 | 0.709 | 0.166 | 0.659 | 0.766 |
| 53 | RM469 | 3 | 0.620 | 0.523 | 0.041 | 0.451 | 0.921 |
| 54 | RM6547 | 3 | 0.866 | 0.239 | 0.016 | 0.226 | 0.931 |
| 55 | RM152 | 4 | 0.508 | 0.628 | 0.016 | 0.565 | 0.973 |
| 56 | RM148 | 2 | 0.675 | 0.438 | 0.083 | 0.342 | 0.811 |
| 57 | RM421 | 3 | 0.458 | 0.630 | 0 | 0.554 | 1 |
| 58 | RM2634 | 3 | 0.379 | 0.658 | 0.025 | 0.583 | 0.962 |
| 59 | RM248 | 4 | 0.345 | 0.732 | 0.116 | 0.683 | 0.841 |
| 60 | RM7179 | 5 | 0.325 | 0.765 | 0.358 | 0.726 | 0.534 |
| 61 | RM215 | 3 | 0.616 | 0.490 | 0.016 | 0.392 | 0.966 |
| 62 | RM324 | 4 | 0.541 | 0.635 | 0.158 | 0.590 | 0.752 |
| 63 | RM317 | 3 | 0.725 | 0.403 | 0 | 0.328 | 1 |
| 64 | RM174 | 3 | 0.508 | 0.620 | 0.066 | 0.550 | 0.893 |
| 65 | RM556 | 3 | 0.841 | 0.278 | 0.033 | 0.260 | 0.881 |
| 66 | RM257 | 4 | 0.408 | 0.663 | 0.233 | 0.594 | 0.650 |
| 67 | RM502 | 3 | 0.808 | 0.318 | 0 | 0.281 | 1 |
| 68 | RM331 | 4 | 0.483 | 0.663 | 0.058 | 0.611 | 0.912 |
| 69 | RM403 | 4 | 0.595 | 0.570 | 0.083 | 0.515 | 0.854 |
| 70 | RM309 | 3 | 0.695 | 0.460 | 0.025 | 0.405 | 0.946 |
| 71 | RM6641 | 3 | 0.566 | 0.582 | 0 | 0.516 | 1 |
| 72 | RM3 | 3 | 0.383 | 0.662 | 0.033 | 0.588 | 0.950 |
| 73 | RM594 | 3 | 0.587 | 0.557 | 0.008 | 0.488 | 0.985 |
| 74 | RM3392 | 4 | 0.504 | 0.615 | 0.108 | 0.544 | 0.825 |
| 75 | RM1278 | 3 | 0.783 | 0.360 | 0.066 | 0.328 | 0.816 |
| 76 | RM168 | 3 | 0.625 | 0.509 | 0.15 | 0.431 | 0.707 |
| 77 | RM3375 | 3 | 0.566 | 0.576 | 0.033 | 0.506 | 0.942 |
| 78 | RM282 | 3 | 0.725 | 0.436 | 0 | 0.395 | 1 |
| 79 | RM26632 | 4 | 0.362 | 0.700 | 0.158 | 0.644 | 0.775 |
| 80 | RM1341 | 3 | 0.612 | 0.528 | 0.025 | 0.455 | 0.953 |
| 81 | RM4112 | 3 | 0.487 | 0.622 | 0.158 | 0.548 | 0.747 |
| 82 | RM20377 | 4 | 0.770 | 0.369 | 0.066 | 0.326 | 0.820 |
| 83 | RM210 | 5 | 0.362 | 0.734 | 0.7 | 0.686 | 0.050 |
| 84 | RM218 | 4 | 0.583 | 0.584 | 0.033 | 0.531 | 0.943 |
| 85 | RM494 | 5 | 0.383 | 0.716 | 0.025 | 0.669 | 0.965 |
| 86 | RM336 | 5 | 0.383 | 0.710 | 0.091 | 0.660 | 0.872 |
| 87 | RM3475 | 4 | 0.45 | 0.656 | 0.041 | 0.590 | 0.937 |
| 88 | RM480 | 4 | 0.537 | 0.617 | 0.025 | 0.560 | 0.959 |
| 89 | RM566 | 4 | 0.433 | 0.656 | 0.016 | 0.591 | 0.974 |
| 90 | RM11701 | 3 | 0.641 | 0.471 | 0 | 0.374 | 1 |
| 91 | RM220 | 6 | 0.358 | 0.745 | 0.183 | 0.703 | 0.755 |
| 92 | RM488 | 6 | 0.320 | 0.749 | 0.191 | 0.708 | 0.746 |
| 93 | RM6374 | 6 | 0.337 | 0.771 | 0.075 | 0.737 | 0.903 |
| 94 | RM233 | 5 | 0.35 | 0.727 | 0.233 | 0.680 | 0.681 |
| 95 | RM112 | 3 | 0.875 | 0.222 | 0 | 0.203 | 1 |
| 96 | RM13600 | 4 | 0.479 | 0.662 | 0.1 | 0.607 | 0.850 |
| 97 | RM495 | 3 | 0.6 | 0.559 | 0.033 | 0.498 | 0.940 |
| 98 | RM493 | 7 | 0.283 | 0.812 | 0.558 | 0.787 | 0.316 |
| 99 | RM444 | 5 | 0.320 | 0.772 | 0.158 | 0.736 | 0.796 |
| 100 | RM468 | 3 | 0.770 | 0.378 | 0.025 | 0.346 | 0.934 |
| 101 | RM6054 | 3 | 0.925 | 0.141 | 0.016 | 0.136 | 0.883 |
| 102 | RM509 | 3 | 0.758 | 0.394 | 0 | 0.359 | 1 |
| 103 | RM5638 | 6 | 0.612 | 0.587 | 0.133 | 0.557 | 0.774 |
| 104 | RM8044 | 6 | 0.279 | 0.760 | 0.233 | 0.720 | 0.695 |
| 105 | RM8271 | 5 | 0.404 | 0.723 | 0.133 | 0.679 | 0.817 |
| 106 | RM171 | 4 | 0.516 | 0.633 | 0.058 | 0.575 | 0.908 |
| 107 | RM16686 | 3 | 0.416 | 0.655 | 0 | 0.581 | 1 |
| 108 | RM434 | 4 | 0.566 | 0.594 | 0.025 | 0.537 | 0.958 |
| 109 | RM6091 | 4 | 0.816 | 0.318 | 0 | 0.298 | 1 |
| 110 | RM209 | 4 | 0.541 | 0.612 | 0 | 0.552 | 1 |
| 111 | RM245 | 4 | 0.583 | 0.577 | 0 | 0.518 | 1 |
| 112 | RM1089 | 4 | 0.416 | 0.636 | 0.066 | 0.565 | 0.896 |
| 113 | RM228 | 4 | 0.625 | 0.544 | 0.191 | 0.490 | 0.650 |
| 114 | RM401 | 3 | 0.754 | 0.397 | 0.058 | 0.359 | 0.854 |
| 115 | RM11 | 3 | 0.462 | 0.590 | 0.008 | 0.502 | 0.985 |
| 116 | RM3351 | 3 | 0.583 | 0.517 | 0 | 0.419 | 1 |
| 117 | RM5749 | 3 | 0.587 | 0.504 | 0.025 | 0.399 | 0.950 |
| 118 | RM335 | 2 | 0.720 | 0.402 | 0.075 | 0.321 | 0.815 |
| 119 | RM144 | 3 | 0.587 | 0.515 | 0.158 | 0.418 | 0.695 |
| 120 | RM300 | 3 | 0.866 | 0.237 | 0.016 | 0.221 | 0.930 |
| 121 | RM1132 | 4 | 0.358 | 0.724 | 0.033 | 0.673 | 0.954 |
| 122 | RM400 | 4 | 0.366 | 0.717 | 0.466 | 0.664 | 0.353 |
| 123 | RM471 | 3 | 0.8 | 0.337 | 0 | 0.309 | 1 |
| 124 | RM243 | 3 | 0.575 | 0.553 | 0.016 | 0.475 | 0.970 |
| 125 | RM467 | 3 | 0.558 | 0.575 | 0 | 0.501 | 1 |
| 126 | RM564 | 4 | 0.45 | 0.599 | 0.1 | 0.515 | 0.834 |
| 127 | RM8007 | 3 | 0.766 | 0.384 | 0 | 0.352 | 1 |
| 128 | RM441 | 4 | 0.475 | 0.627 | 0.566 | 0.557 | 0.100 |
| 129 | RM518 | 3 | 0.541 | 0.537 | 0 | 0.437 | 1 |
| 130 | RM253 | 4 | 0.554 | 0.594 | 0.083 | 0.529 | 0.860 |
| 131 | RM274 | 3 | 0.666 | 0.476 | 0 | 0.405 | 1 |
| 132 | RM242 | 4 | 0.575 | 0.590 | 0.016 | 0.535 | 0.972 |
| 133 | RM3231 | 4 | 0.345 | 0.703 | 0.65 | 0.644 | 0.079 |
| 134 | RM5687 | 4 | 0.416 | 0.687 | 0.65 | 0.629 | 0.058 |
| 135 | RM5626 | 3 | 0.583 | 0.511 | 0.733 | 0.410 | -0.429 |
| 136 | RM452 | 3 | 0.475 | 0.618 | 0 | 0.540 | 1 |
|  | **Mean** | **3.7** | **0.561** | **0.554** | **0.114** | **0.495** | **0.795** |
