# Supplementary material for: Association mapping for protein, total soluble sugars, starch, amylose and chlorophyll content in rice
Source: BMC Plant Biol. 2022 Dec 29;22:620. doi: 10.1186/s12870-022-04015-8 (PMC9801606; doi:10.1186/s12870-022-04015-8)
Supplement: Supplementary file 6 — Additional file 6: Supplementary Table 4. Genetic structure ancestry value at K = 3 and classification of the panel population containing 120 landraces based on the biochemical traits. [file 12870_2022_4015_MOESM6_ESM.docx]

Supplementary Table 4. Genetic structure ancestry value at K=3 and classification of the panel population containing 120 landraces based on the biochemical traits.

| Sl. No. | Name of the germplasm | Inferred ancestry value at K=3 | | | | Classification of the germplasm lines based on the biochemical traits | | | | | |
| --- | --- | --- | --- | --- | --- | --- | --- | --- | --- | --- | --- |
|  |  | Q1 | Q2 | Q3 | SP | Chla | Chlb | Starch | Amylose | TP | TSS |
| 1 | Kalimekri77-5 | 0.002 | 0.008 | 0.989 | 3 | VL | VL | H | M | M | H |
| 2 | PMK2 | 0.003 | 0.018 | 0.98 | 3 | VL | L | M | M | L | L |
| 3 | TKM10 | 0.001 | 0.002 | 0.997 | 3 | VL | L | H | H | VL | M |
| 4 | Belimuruduga | 0.002 | 0.002 | 0.996 | 3 | VL | L | H | M | M | VL |
| 5 | Koompallai | 0.001 | 0.001 | 0.998 | 3 | H | H | M | M | M | M |
| 6 | Karinellu | 0.002 | 0.002 | 0.996 | 3 | VL | M | H | M | H | H |
| 7 | Gouri | 0.002 | 0.001 | 0.998 | 3 | VH | VH | M | H | VL | H |
| 8 | Chitapa | 0.004 | 0.006 | 0.989 | 3 | VH | VH | VH | M | H | VH |
| 9 | Bilipandya | 0.002 | 0.001 | 0.997 | 3 | VH | VH | M | M | VL | H |
| 10 | Jayapadma | 0.001 | 0.001 | 0.998 | 3 | VH | VH | VH | M | H | VL |
| 11 | Jira | 0.008 | 0.006 | 0.986 | 3 | VH | VH | H | H | VL | VL |
| 12 | Sonamasuri | 0.022 | 0.001 | 0.976 | 3 | VH | VH | M | M | VL | L |
| 13 | Kanakchampa | 0.982 | 0.015 | 0.003 | 1 | VL | VL | M | M | H | L |
| 14 | Laxmibilash | 0.432 | 0.565 | 0.003 | A | M | VH | M | M | H | L |
| 15 | Lalgundi | 0.991 | 0.005 | 0.003 | 1 | VL | VL | M | H | VH | L |
| 16 | Magra | 0.996 | 0.003 | 0.002 | 1 | VL | VL | M | M | H | L |
| 17 | Gondiachampeisiali | 0.996 | 0.002 | 0.002 | 1 | L | VL | H | M | H | L |
| 18 | Gandhakasala | 0.004 | 0.992 | 0.003 | 2 | VL | VL | M | M | H | M |
| 19 | D1 | 0.945 | 0.011 | 0.044 | 1 | VL | VL | H | M | VH | L |
| 20 | Mahamaga | 0.949 | 0.002 | 0.05 | 1 | L | L | H | M | VH | VL |
| 21 | Jhingesal | 0.998 | 0.001 | 0.001 | 1 | M | M | H | H | L | L |
| 22 | Gochi | 0.931 | 0.008 | 0.06 | 1 | VL | L | H | M | H | H |
| 23 | Chatuimuchi | 0.001 | 0.998 | 0.001 | 2 | M | H | M | H | H | L |
| 24 | Dudhamani | 0.94 | 0.002 | 0.059 | 1 | L | M | M | H | M | L |
| 25 | Mahipaljeera | 0.894 | 0.007 | 0.1 | 1 | VH | M | H | M | H | H |
| 26 | Batachudi | 0.98 | 0.012 | 0.008 | 1 | H | M | M | M | L | VH |
| 27 | Salati | 0.996 | 0.001 | 0.003 | 1 | M | L | H | M | H | H |
| 28 | Kusumal | 0.949 | 0.009 | 0.041 | 1 | H | L | M | M | M | VH |
| 29 | Phongangangamphou | 0.995 | 0.004 | 0.001 | 1 | M | M | VL | M | H | L |
| 30 | Langmanbu | 0.998 | 0.001 | 0.001 | 1 | H | VH | M | M | VH | L |
| 31 | Moirangphon | 0.967 | 0.03 | 0.003 | 1 | M | H | M | M | M | L |
| 32 | Chakhaosimpak | 0.988 | 0.009 | 0.003 | 1 | L | M | M | H | M | M |
| 33 | Kartiksal | 0.998 | 0.001 | 0.002 | 1 | VH | VH | VH | M | VH | VL |
| 34 | Champalidhan | 0.997 | 0.001 | 0.002 | 1 | VL | VL | M | M | H | L |
| 35 | Ahimachutki | 0.96 | 0.017 | 0.023 | 1 | VL | L | H | M | H | VH |
| 36 | Ampang | 0.994 | 0.001 | 0.005 | 1 | L | L | L | H | H | VH |
| 37 | Latamahu | 0.996 | 0.001 | 0.003 | 1 | VL | VL | M | M | M | VL |
| 38 | Kundadhan | 0.993 | 0.001 | 0.006 | 1 | L | L | VH | M | H | H |
| 39 | Karpurkanti | 0.002 | 0.998 | 0.001 | 2 | M | VL | M | M | L | VL |
| 40 | Kantakaamala | 0.683 | 0.218 | 0.099 | A | M | VL | VH | L | M | M |
| 41 | Jyothi | 0.998 | 0.001 | 0.001 | 1 | L | L | H | M | VH | L |
| 42 | Marathondi | 0.507 | 0.451 | 0.043 | A | L | M | H | M | VH | M |
| 43 | Vachaw | 0.959 | 0.039 | 0.002 | 1 | L | L | M | M | H | M |
| 44 | Adira-3 | 0.601 | 0.396 | 0.003 | A | L | M | H | M | H | H |
| 45 | Adira-1 | 0.628 | 0.355 | 0.017 | A | L | M | VH | M | VH | L |
| 46 | Bharati | 0.997 | 0.002 | 0.001 | 1 | VL | L | VH | M | VH | M |
| 47 | Shayam | 0.003 | 0.996 | 0.001 | 2 | M | M | M | M | H | VL |
| 48 | Jhagrikartik | 0.989 | 0.002 | 0.009 | 1 | VL | VL | M | M | H | L |
| 49 | Liktimachi | 0.996 | 0.001 | 0.003 | 1 | H | L | VL | M | H | L |
| 50 | Chudi | 0.998 | 0.001 | 0.002 | 1 | L | H | H | M | VH | M |
| 51 | Jhitikuji | 0.996 | 0.001 | 0.003 | 1 | VH | M | M | H | M | M |
| 52 | Pondremunduria | 0.993 | 0.004 | 0.003 | 1 | L | M | H | M | VH | M |
| 53 | Phoudum | 0.997 | 0.001 | 0.002 | 1 | L | M | M | M | M | M |
| 54 | Taothali | 0.983 | 0.014 | 0.003 | 1 | L | M | M | H | M | M |
| 55 | Mayangkhang-I | 0.919 | 0.003 | 0.078 | 1 | L | VH | VH | L | VH | L |
| 56 | Aujari | 0.967 | 0.008 | 0.025 | 1 | L | VH | L | M | H | L |
| 57 | Chingforechokua | 0.007 | 0.991 | 0.002 | 2 | VL | VL | M | H | H | L |
| 58 | Tilibora | 0.98 | 0.002 | 0.019 | 1 | VL | VL | M | M | H | VL |
| 59 | Kanaimuluk | 0.908 | 0.003 | 0.088 | 1 | VL | VL | M | H | M | L |
| 60 | Mikirahu | 0.995 | 0.002 | 0.002 | 1 | VL | VL | L | M | H | VH |
| 61 | Pratao | 0.008 | 0.035 | 0.957 | 3 | VL | L | VH | M | H | M |
| 62 | Aditya | 0.002 | 0.172 | 0.826 | 3 | VL | L | H | L | M | M |
| 63 | Noorthipathu | 0.001 | 0.001 | 0.998 | 3 | L | L | VH | M | VH | H |
| 64 | Tulasi | 0.004 | 0.002 | 0.994 | 3 | VH | VH | VH | M | VH | L |
| 65 | MDU-5 | 0.004 | 0.001 | 0.995 | 3 | VL | L | M | M | M | VL |
| 66 | Manavari | 0.002 | 0.001 | 0.997 | 3 | L | L | VH | M | M | M |
| 67 | Pandya | 0.009 | 0.001 | 0.99 | 3 | VH | VH | VH | M | M | VH |
| 68 | Badra | 0.002 | 0.001 | 0.997 | 3 | VH | VH | VH | M | H | L |
| 69 | Kalame | 0.001 | 0.717 | 0.282 | A | H | VH | M | M | M | VL |
| 70 | Lusai | 0.014 | 0.01 | 0.976 | 3 | VH | VH | M | M | VL | L |
| 71 | Malbar | 0.001 | 0.008 | 0.991 | 3 | VH | VH | VH | M | M | VH |
| 72 | Bilijaya | 0.004 | 0.008 | 0.988 | 3 | VH | VH | M | H | L | L |
| 73 | Magura-s | 0.916 | 0.082 | 0.002 | 1 | VL | VL | L | M | H | VL |
| 74 | Kaniar | 0.981 | 0.014 | 0.004 | 1 | L | VL | H | M | VH | M |
| 75 | Balisaralaktimachi-k | 0.994 | 0.003 | 0.003 | 1 | VL | VL | L | M | M | L |
| 76 | Landi | 0.997 | 0.002 | 0.001 | 1 | H | L | H | M | H | M |
| 77 | Chinamal | 0.985 | 0.014 | 0.001 | 1 | L | VL | M | H | M | L |
| 78 | Sreyas | 0.996 | 0.002 | 0.002 | 1 | VL | VL | H | M | VH | VL |
| 79 | Pk-21 | 0.984 | 0.001 | 0.015 | 1 | VL | L | H | M | VH | H |
| 80 | Jaya | 0.992 | 0.001 | 0.007 | 1 | VL | VL | L | VH | H | VL |
| 81 | Cheruvirippu | 0.995 | 0.001 | 0.003 | 1 | VL | L | H | M | VH | L |
| 82 | Sugandha-2 | 0.002 | 0.997 | 0.001 | 2 | L | M | M | M | M | L |
| 83 | Uttarbangalocal-9 | 0.899 | 0.002 | 0.099 | 1 | VL | L | L | H | M | VL |
| 84 | Palinadhan-1 | 0.412 | 0.366 | 0.222 | A | VL | L | M | H | M | L |
| 85 | BodiKaberi | 0.889 | 0.083 | 0.028 | 1 | M | L | H | M | M | VH |
| 86 | Barda | 0.973 | 0.024 | 0.003 | 1 | H | L | M | H | M | H |
| 87 | Kaberi | 0.976 | 0.007 | 0.017 | 1 | M | L | M | H | M | M |
| 88 | Kakudimanji | 0.993 | 0.001 | 0.006 | 1 | M | L | L | H | L | M |
| 89 | Phourrel | 0.994 | 0.002 | 0.004 | 1 | VH | VH | M | M | H | L |
| 90 | Kakchengphou | 0.827 | 0.007 | 0.167 | 1 | VL | L | M | M | VH | L |
| 91 | Chingphou | 0.992 | 0.001 | 0.007 | 1 | L | VH | VL | M | H | L |
| 92 | Phoaujaarangbele | 0.994 | 0.001 | 0.005 | 1 | L | VH | M | M | M | L |
| 93 | Turnaianganba | 0.995 | 0.003 | 0.001 | 1 | M | H | M | H | M | H |
| 94 | Memabalbok | 0.798 | 0.195 | 0.007 | 1 | L | L | M | H | H | VL |
| 95 | Mimagisim | 0.002 | 0.997 | 0.001 | 2 | M | VL | L | H | H | H |
| 96 | Mimahambel | 0.9 | 0.002 | 0.098 | 1 | M | M | M | L | H | H |
| 97 | Latachaunri | 0.993 | 0.002 | 0.005 | 1 | L | VL | VH | M | H | M |
| 98 | Champaeisiali | 0.993 | 0.004 | 0.003 | 1 | VL | VL | VH | M | H | H |
| 99 | Kathidhan | 0.894 | 0.004 | 0.102 | 1 | L | L | H | M | M | L |
| 100 | kapanthi | 0.306 | 0.352 | 0.342 | A | L | VL | VL | VH | L | M |
| 101 | Kantakapura | 0.997 | 0.002 | 0.001 | 1 | M | VL | VH | M | H | H |
| 102 | Ezhoml-2 | 0.998 | 0.001 | 0.001 | 1 | L | L | H | M | VH | L |
| 103 | Kozhivalan | 0.997 | 0.001 | 0.002 | 1 | L | L | H | M | VH | M |
| 104 | PK6 | 0.982 | 0.014 | 0.004 | 1 | VL | L | H | M | VH | L |
| 105 | Adira-2 | 0.997 | 0.001 | 0.002 | 1 | M | M | H | M | VH | H |
| 106 | Joha | 0.997 | 0.002 | 0.001 | 1 | VL | VL | M | M | H | VL |
| 107 | Basumati-B | 0.124 | 0.873 | 0.004 | 2 | M | VL | M | M | M | M |
| 108 | Dadghani | 0.991 | 0.003 | 0.006 | 1 | M | M | M | M | H | L |
| 109 | Baranga | 0.996 | 0.003 | 0.001 | 1 | M | L | VH | M | VH | M |
| 110 | Radhabati | 0.997 | 0.001 | 0.002 | 1 | H | M | M | M | M | H |
| 111 | Tikichudi | 0.993 | 0.005 | 0.001 | 1 | L | H | H | M | H | M |
| 112 | Lalmunduria | 0.998 | 0.001 | 0.001 | 1 | H | M | H | M | H | L |
| 113 | Changli | 0.993 | 0.005 | 0.003 | 1 | VH | VH | M | H | H | M |
| 114 | Mayangkhang-II | 0.998 | 0.001 | 0.001 | 1 | M | H | L | M | H | L |
| 115 | Moiranghouanganba | 0.994 | 0.001 | 0.005 | 1 | L | VH | M | M | M | L |
| 116 | Kabokphou | 0.982 | 0.005 | 0.014 | 1 | M | H | M | M | H | L |
| 117 | Manipurlocal | 0.995 | 0.002 | 0.003 | 1 | VL | L | M | M | M | L |
| 118 | ManoharSali | 0.957 | 0.002 | 0.042 | 1 | VL | VL | M | H | M | L |
| 119 | Bengalijoha | 0.996 | 0.002 | 0.003 | 1 | VL | VL | M | H | H | L |
| 120 | Anapachidhan | 0.991 | 0.002 | 0.007 | 1 | L | L | M | H | M | H |

Chl a: Chlorophyll a content; Chl b: Chlorophyll b content; Starch: Starch content; Amylose: Amylose content; TP: Total protein content; TSS: Total soluble sugars content; VL: Very low; L: Low; M: Medium; H: High; VH: Very high
