# Supplementary material for: Association mapping for protein, total soluble sugars, starch, amylose and chlorophyll content in rice
Source: BMC Plant Biol. 2022 Dec 29;22:620. doi: 10.1186/s12870-022-04015-8 (PMC9801606; doi:10.1186/s12870-022-04015-8)
Supplement: Supplementary file 7 — Additional file 7: Supplementary Table 5. Significant marker-trait associations detected for chlorophyll a, chlorophyll b, starch, amylose, total protein and total soluble sugars by GLM approach at p < 0.01. [file 12870_2022_4015_MOESM7_ESM.docx]

**Supplementary Table 5**. Significant marker-trait associations detected for chlorophyll a, chlorophyll b, starch, amylose, total protein and total soluble sugars by GLM approach at p<0.01.

| **Sl No** | **Trait** | **Marker** | **Chr#** | **Position** | **marker_F** | **marker_p** | **marker_r^2^** |
| --- | --- | --- | --- | --- | --- | --- | --- |
| 1 | Chl a | RM328 | 9 | 26 | 9.28788 | 0.00286 | 0.06583 |
| 2 | Chl a | RM337 | 8 | 27 | 8.76574 | 0.00372 | 0.06239 |
| 3 | Chl a | RM3701 | 11 | 56 | 8.33826 | 0.00463 | 0.05955 |
| 4 | Chl a | RM201 | 9 | 68 | 8.63773 | 0.00397 | 0.06155 |
| 5 | Chl a | RM201 | 9 | 69 | 8.44091 | 0.00439 | 0.06024 |
| 6 | Chl a | RM1347 | 2 | 82 | 12.06469 | 7.23E-04 | 0.08366 |
| 7 | Chl a | RM5793 | 7 | 107 | 8.2867 | 0.00476 | 0.05921 |
| 8 | Chl a | RM405 | 5 | 109 | 18.22659 | 4.03E-05 | 0.12059 |
| 9 | Chl a | RM7179 | 6 | 159 | 6.92646 | 0.00965 | 0.05004 |
| 10 | Chl a | RM3392 | 3 | 194 | 7.70055 | 0.00644 | 0.05528 |
| 11 | Chl a | RM4112 | 11 | 209 | 7.35857 | 0.00769 | 0.05297 |
| 12 | Chl a | RM494 | 6 | 222 | 8.45267 | 0.00437 | 0.06032 |
| 13 | Chl a | RM1132 | 7 | 331 | 7.51606 | 0.00708 | 0.05404 |
| 14 | Chl a | RM441 | 11 | 349 | 8.36543 | 0.00457 | 0.05974 |
| 15 | Chl a | RM3231 | 8 | 363 | 16.6411 | 8.32E-05 | 0.11142 |
| 16 | Chl b | RM328 | 9 | 26 | 6.90263 | 0.00977 | 0.04862 |
| 17 | Chl b | RM337 | 8 | 27 | 8.08167 | 0.00529 | 0.05638 |
| 18 | Chl b | RM22034 | 7 | 56 | 12.16286 | 6.90E-04 | 0.08215 |
| 19 | Chl b | RM440 | 5 | 67 | 11.55526 | 9.27E-04 | 0.07842 |
| 20 | Chl b | RM201 | 9 | 68 | 8.13135 | 0.00515 | 0.05671 |
| 21 | Chl b | RM201 | 9 | 69 | 8.68381 | 0.00388 | 0.06029 |
| 22 | Chl b | RM3735 | 4 | 80 | 9.29523 | 0.00285 | 0.06422 |
| 23 | Chl b | RM1347 | 2 | 82 | 10.98502 | 0.00123 | 0.07488 |
| 24 | Chl b | RM5793 | 7 | 107 | 8.56924 | 0.00412 | 0.05955 |
| 25 | Chl b | RM405 | 5 | 109 | 15.57377 | 1.36E-04 | 0.10246 |
| 26 | Chl b | RM5436 | 7 | 136 | 7.80936 | 0.00608 | 0.0546 |
| 27 | Chl b | RM3392 | 3 | 194 | 7.10951 | 0.00876 | 0.04999 |
| 28 | Chl b | RM403 | 1 | 331 | 7.01103 | 0.00923 | 0.04934 |
| 29 | Chl b | RM168 | 3 | 349 | 8.84863 | 0.00357 | 0.06135 |
| 30 | Chl b | RM3231 | 8 | 363 | 15.11793 | 1.69E-04 | 0.09981 |
| 31 | Starch | RM472 | 1 | 40 | 6.96737 | 0.00944 | 0.05221 |
| 32 | Starch | RM3701 | 11 | 48 | 9.07463 | 0.00318 | 0.06686 |
| 33 | Starch | RM7179 | 6 | 161 | 11.42779 | 9.86E-04 | 0.08264 |
| 34 | Starch | RM3 | 6 | 190 | 9.03862 | 0.00324 | 0.06661 |
| 35 | Starch | RM20377 | 6 | 212 | 10.02303 | 0.00198 | 0.07329 |
| 36 | Starch | RM6374 | 2 | 249 | 11.93771 | 7.69E-04 | 0.08598 |
| 37 | Amylose | RM3701 | 11 | 48 | 13.21964 | 4.14E-04 | 0.09824 |
| 38 | Amylose | RM315 | 1 | 92 | 7.41586 | 0.00746 | 0.0577 |
| 39 | Amylose | RM167 | 11 | 123 | 10.03053 | 0.00197 | 0.07642 |
| 40 | Amylose | RM6091 | 11 | 304 | 16.06201 | 1.09E-04 | 0.11679 |
| 41 | TP | RM472 | 1 | 40 | 10.99408 | 0.00122 | 0.06908 |
| 42 | TP | RM472 | 1 | 41 | 9.31562 | 0.00282 | 0.05932 |
| 43 | TP | RM223 | 8 | 60 | 7.44781 | 0.00734 | 0.04815 |
| 44 | TP | RM167 | 11 | 121 | 9.03784 | 0.00324 | 0.05768 |
| 45 | TP | RM324 | 2 | 167 | 7.92778 | 0.00572 | 0.05105 |
| 46 | TP | RM566 | 8 | 234 | 8.23343 | 0.00489 | 0.05289 |
| 47 | TP | RM220 | 1 | 240 | 10.52602 | 0.00154 | 0.06639 |
| 48 | TP | RM5638 | 1 | 282 | 10.91588 | 0.00127 | 0.06864 |
| 49 | TP | RM253 | 6 | 355 | 9.72444 | 0.00229 | 0.06172 |
| 50 | TSS | RM5310 | 1 | 2 | 8.15918 | 0.00508 | 0.06459 |
| 51 | TSS | RM247 | 12 | 23 | 8.46122 | 0.00435 | 0.06682 |
| 52 | TSS | RM247 | 12 | 24 | 7.85764 | 0.00593 | 0.06235 |
| 53 | TSS | RM337 | 8 | 27 | 7.46984 | 0.00726 | 0.05946 |
| 54 | TSS | RM248 | 7 | 157 | 24.15956 | 2.94E-06 | 0.16942 |
| 55 | TSS | RM566 | 8 | 236 | 8.82874 | 0.00361 | 0.06952 |
| 56 | TSS | RM434 | 9 | 300 | 8.49713 | 0.00427 | 0.06708 |
| 57 | TSS | RM434 | 9 | 301 | 9.63243 | 0.0024 | 0.07536 |
| 58 | TSS | RM3351 | 5 | 321 | 7.0358 | 0.00911 | 0.05621 |
| 59 | TSS | RM3231 | 8 | 363 | 7.85568 | 0.00594 | 0.06234 |
| 60 | TSS | RM5687 | 4 | 364 | 9.19841 | 0.00299 | 0.07221 |

Chl a: Chlorophyll a content; Chl b: Chlorophyll b content; Starch: Starch content; Amylose: Amylose content; TP: Total protein content; TSS: Total soluble sugars content
