# Supplementary material for: Association mapping for protein, total soluble sugars, starch, amylose and chlorophyll content in rice
Source: BMC Plant Biol. 2022 Dec 29;22:620. doi: 10.1186/s12870-022-04015-8 (PMC9801606; doi:10.1186/s12870-022-04015-8)
Supplement: Supplementary file 8 — Additional file 8: Supplementary Table 6. Significant marker-trait associations detected for chlorophyll a, chlorophyll b, starch, amylose, total protein and total soluble sugars by MLM approach at p < 0.01. [file 12870_2022_4015_MOESM8_ESM.docx]

**Supplementary Table 6**. Significant marker-trait associations detected for chlorophyll a, chlorophyll b, starch, amylose, total protein and total soluble sugars by MLM approach at p<0.01

| Sl No | Traits | Markers | Chr# | Position | F value | p-value | Marker_ r2 |
| --- | --- | --- | --- | --- | --- | --- | --- |
| 1 | Chl a | RM1347 | 2 | 82 | 8.85234 | 0.00356 | 0.07251 |
| 2 | Chl a | RM405 | 5 | 109 | 7.46088 | 0.00729 | 0.06111 |
| 3 | Chl a | RM401 | 4 | 318 | 7.83032 | 0.00602 | 0.06414 |
| 4 | Chl a | RM3231 | 8 | 363 | 11.98915 | 7.50E-04 | 0.0982 |
| 5 | Chl b | RM440 | 5 | 67 | 7.76369 | 0.00623 | 0.0628 |
| 6 | Chl b | RM5436 | 7 | 136 | 6.86045 | 0.00999 | 0.05549 |
| 7 | Chl b | RM401 | 4 | 318 | 8.53913 | 0.00418 | 0.06907 |
| 8 | Chl b | RM3231 | 8 | 363 | 9.27211 | 0.00288 | 0.075 |
| 9 | Starch | RM3701 | 11 | 48 | 7.79603 | 0.00613 | 0.06645 |
| 10 | Starch | RM20377 | 6 | 212 | 8.32763 | 0.00466 | 0.07099 |
| 11 | Starch | RM6374 | 2 | 247 | 7.48808 | 0.00719 | 0.06383 |
| 12 | Starch | RM6374 | 2 | 249 | 7.03173 | 0.00913 | 0.05994 |
| 13 | Amylose | RM3701 | 11 | 48 | 13.32049 | 3.95E-04 | 0.11376 |
| 14 | Amylose | RM315 | 1 | 92 | 8.42245 | 0.00444 | 0.07193 |
| 15 | Amylose | RM167 | 11 | 123 | 9.27197 | 0.00288 | 0.07918 |
| 16 | Amylose | RM1341 | 11 | 208 | 6.92551 | 0.00965 | 0.05914 |
| 17 | Amylose | RM6091 | 11 | 304 | 12.86845 | 4.91E-04 | 0.1099 |
| 18 | TP | RM566 | 8 | 234 | 7.10636 | 0.00878 | 0.05832 |
| 19 | TP | RM220 | 1 | 240 | 7.08199 | 0.00889 | 0.05812 |
| 20 | TP | RM5638 | 1 | 282 | 8.73341 | 0.00378 | 0.07168 |
| 21 | TP | RM253 | 6 | 355 | 7.99151 | 0.00554 | 0.06559 |
| 22 | TSS | RM247 | 12 | 23 | 7.12111 | 0.00871 | 0.06081 |
| 23 | TSS | RM337 | 8 | 27 | 8.09955 | 0.00524 | 0.06916 |
| 24 | TSS | RM340 | 6 | 34 | 6.92142 | 0.00967 | 0.0591 |
| 25 | TSS | RM248 | 7 | 157 | 14.13975 | 2.67E-04 | 0.12074 |
| 26 | TSS | RM566 | 8 | 236 | 7.78093 | 0.00617 | 0.06644 |

Chla: Chlorophyll a content; Chlb: Chlorophyll b content; Starch: Starch content; Amylose: Amylose content; TP: Total protein content; TSS: Total soluble sugars content
